# The Increased Burden of Rare Variants in Four Matrix Metalloproteinase-Related Genes in Childhood Glaucoma Suggests a Complex Genetic Inheritance of the Disease

**DOI:** 10.3390/ijms25115757

**Published:** 2024-05-25

**Authors:** Angel Tevar, José-Daniel Aroca-Aguilar, Juan-Manuel Bonet-Fernández, Raquel Atienzar-Aroca, Ezequiel Campos-Mollo, Carmen Méndez-Hernández, Laura Morales-Fernández, Iñaki Leal Palmer, Miguel Coca-Prados, Jose-Maria Martinez-de-la-Casa, Julian Garcia-Feijoo, Julio Escribano

**Affiliations:** 1 Área de Genética, Facultad de Medicina de Albacete, Universidad de Castilla-La Mancha, 02006 Albacete, Spain; angel.tevar@uclm.es (A.T.); josedaniel.aroca@uclm.es (J.-D.A.-A.); juanm.bonet@uclm.es (J.-M.B.-F.); raquelatiaro@hotmail.com (R.A.-A.); 2 Biomedicine Institute, Universidad de Castilla-La Mancha, 02006 Albacete, Spain; 3 Cooperative Research Network on Age-Related Ocular Pathology, Visual and Life Quality (OFTARED), Instituto de Salud Carlos III, 28029 Madrid, Spain; ezechiel@hotmail.com (E.C.-M.); cdmendezh@gmail.com (C.M.-H.); lauramoralesfernandez@gmail.com (L.M.-F.); jmmartinezcasa@gmail.com (J.-M.M.-d.-l.-C.); jgarciafeijoo@hotmail.com (J.G.-F.); 4 Servicio de Oftalmología, Hospital Virgen de los Lirios, 03804 Alcoy, Spain; inalepal98@gmail.com; 5 Servicio de Oftalmología, Hospital Clínico San Carlos, 28040 Madrid, Spain; 6 Instituto de Investigación Sanitaria del Hospital Clínico San Carlos, 28040 Madrid, Spain; 7 Instituto de Investigaciones Oftalmológicas Ramón Castroviejo, Universidad Complutense de Madrid, 28040 Madrid, Spain; 8 Department of Ophthalmology and Visual Science, Yale University Medical School, New Haven, CT 06510, USA; miguel.coca-prados@yale.edu

**Keywords:** glaucoma, matrix metalloproteinases, multifactorial inheritance

## Abstract

Childhood glaucoma encompasses congenital and juvenile primary glaucoma, which are heterogeneous, uncommon, and irreversible optic neuropathies leading to visual impairment with a poorly understood genetic basis. Our goal was to identify gene variants associated with these glaucoma types by assessing the mutational burden in 76 matrix metalloproteinase-related genes. We studied 101 childhood glaucoma patients with no identified monogenic alterations using next-generation sequencing. Gene expression was assessed through immunohistochemistry. Functional analysis of selected gene variants was conducted in cultured cells and in zebrafish. Patients presented a higher proportion of rare variants in four metalloproteinase-related genes, including *CPAMD8* and *ADAMTSL4*, compared to controls. ADAMTSL4 protein expression was observed in the anterior segment of both the adult human and zebrafish larvae’s eye, including tissues associated with glaucoma. In HEK-293T cells, expression of four ADAMTSL4 variants identified in this study showed that two variants (p.Arg774Trp and p.Arg98Trp) accumulated intracellularly, inducing endoplasmic reticulum stress. Additionally, overexpressing these ADAMTSL4 variants in zebrafish embryos confirmed partial loss-of-function effects for p.Ser719Leu and p.Arg1083His. Double heterozygous functional suppression of *adamtsl4* and *cpamd8* zebrafish orthologs resulted in reduced volume of both the anterior eye chamber and lens within the chamber, supporting a genetic interaction between these genes. Our findings suggest that accumulation of partial functional defects in matrix metalloproteinase-related genes may contribute to increased susceptibility to early-onset glaucoma and provide further evidence supporting the notion of a complex genetic inheritance pattern underlying the disease.

## 1. Introduction

According to the World Glaucoma Association, childhood primary glaucoma presents before 16–18 years of age and encompasses both primary congenital glaucoma (PCG) and juvenile open-angle glaucoma (JOAG) [1]. These infrequent conditions can be viewed as part of a continuous disease spectrum [2] and represent a clinically diverse group of severe, progressive, and irreversible optic neuropathies characterized by apoptosis of retinal ganglion cells [3]. Untreated childhood glaucoma leads to irreversible visual impairment and blindness. PCG is the most common type of childhood glaucoma [4,5] with varying worldwide incidence, and JOAG has been described to account for approximately 4% of childhood glaucoma cases [6].

Developmental defects of the anterior chamber angle of the eye, known as goniodysgenesis, are believed to play a significant role in the pathogenesis of childhood glaucoma. In PCG, developmental arrest and malformation specifically affecting the trabecular meshwork (TM) and/or Schlemm’s canal (SC) result in trabeculodysgenesis or goniodysgenesis [7,8] JOAG patients present different forms of goniodysgenesis, including abnormally prominent iris processes, high iris insertion or a thick trabecular band that is due to the compact trabecular meshwork [9]. These structural alterations contribute to elevated intraocular pressure (IOP) and subsequent optic nerve damage.

The extracellular matrix (ECM) in the trabecular meshwork (TM) is abnormal in both PCG [8,10,11,12] and JOAG [13]. Physiological regulation of IOP and aqueous humor outflow involve continuous remodeling of the ECM in the TM, which is mediated by various matrix metalloproteinases (MMPs) [14,15], and the closely related A disintegrin and metalloproteinases (ADAMs) and A disintegrin and metalloproteinase with thrombospondin motif (ADAMTSs) [16]. ADAMTSL-like (ADAMTSL) is a second family of proteins that lack catalytic activity, are secreted and are part of the ECM [17,18], with a member implicated in complex alterations that include congenital glaucoma [19].

Childhood glaucoma exhibits primarily a monogenic pattern, although some studies have indicated a complex genetic architecture in a significant proportion of patients [12,20,21,22,23]. Autosomal recessive inheritance is common in primary congenital glaucoma [23,24], while autosomal dominant inheritance is observed in JOAG [25,26]. *CYP1B1* is the major gene involved in recessive PCG [27,28,29,30], and it is functionally disrupted in 18–48% of non-consanguineous European patients [23,31,32,33]. Other genes, such as *LTBP2* [34,35], *MYOC* [36], *FOXC1* [37,38], and *TEK* [39], have also been reported to play a role in this pathology. Although *GPATCH3* [21] and *GUCA1C* [40] have been proposed as candidate genes in PCG, their involvement in the disease remains unconfirmed. *MYOC* is the main gene associated with JOAG glaucoma [41], accounting for 8% to 36% of affected pedigrees [42] and contributing to the disease in 15–20% of European Caucasian patients [43]. *EFEMP1* rare variants have been identified as the cause of JOAG in Filipino [44] and African-American families [45]. This genetic heterogeneity challenges the diagnosis and treatment of patients.

Here, we show an increased frequency of aggregate rare variants in several metalloproteinase-related genes, including *CPAMD8* and *ADAMTSL4*, among childhood glaucoma patients, suggesting that this mutational burden may contribute to disease pathogenicity, as well as the existence of a complex inheritance. In addition, our data show deleterious effects of some of the identified *ADAMTSL4* variants and reveal a functional interaction between *cpamd8* and *adamtsl4* in zebrafish. Finally, these results can be extrapolated to support the idea that other unexplained rare ocular diseases may also involve complex inheritance mechanisms.

## 2. Results

### 2.1. Mutation Burden Analysis

This study included 101 unrelated patients diagnosed with childhood glaucoma, for whom a monogenic alteration could not be identified. Among these patients, 86 had PCG, while 15 had JOAG. The investigation explored the potential combined disruption of 76 MMP-related genes in these patients (Table S1). These genes encode proteins that participate in extracellular matrix (ECM) remodeling of the trabecular meshwork (TM), a process believed to play a key role in the pathogenesis of this type of glaucoma. To that end we carried out a variant burden analysis, in which we filtered heterozygous rare variants (<0.01) with predicted high or moderate functional impact (frameshift, non-sense, missense and donor/acceptor splicing sites). Furthermore, variants with at least 50 reads were compared to controls of European ancestry obtained from the ESP6500 and gnomAD v2.1.1 databases. In total, 338 rare variants were identified in the patients, averaging 4.45 filtered variants per gene (Table S1).

The variant burden analysis in patients across all individual genes revealed significant enrichment of rare variants with predicted high-moderate functional impact in four genes (*ADAMTS2*, *ADAMTS18*, *ADAMTSL4*, and *CPAMD8*), compared to at least one of the two control groups, after applying the extremely conservative Bonferroni correction for multiple testing (*p* < 6.57 × 10^−4^) (Table 1 and Table S1). Additionally, the aggregate variant frequency in these four genes was significantly higher in patients compared to controls (29.20% vs. 9.13–10.03% in controls, *p* < 1 × 10^−15^, Table 1).

**Figure 1:**
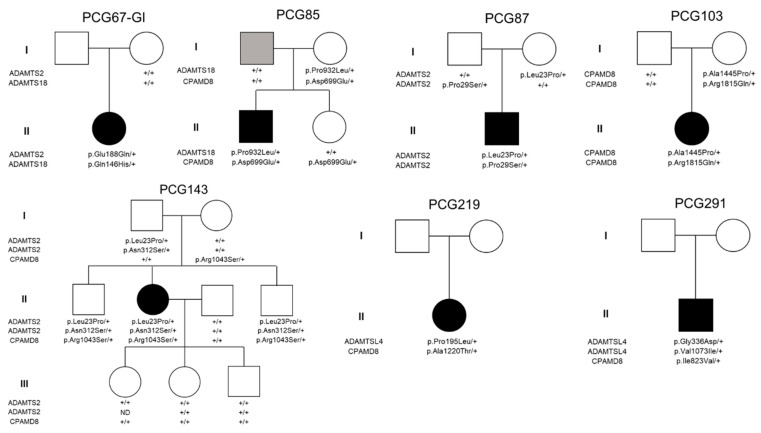
Pedigree and segregation analysis in families of childhood glaucoma patients who carried more than one variant in MMP-related genes. In families PCG219 and PCG291, only proband DNA samples were available for the study. Black and grey symbols indicate childhood glaucoma and primary open-angle glaucoma, respectively. +: wildtype allele.

There was no significant enrichment of rare synonymous variants either across the individual genes (0.99–4.46% vs. 0.97–4.20% in controls, *p* = 0.27–0.99, Table 1) or in the aggregate set of four genes (8.42% vs. 9.05–9.83% in controls, *p* = 0.57–0.63, Table 1). This finding indicates the specificity of the identified association. Therefore, patients with childhood glaucoma exhibited a higher likelihood of carrying rare variants in these MMP-related genes compared to the two control groups. This association was observed both when considering the genes individually, with odds ratios (OR) ranging from 1.77–2.75 for *CPAMD8* to 4.49–4.70 for *ADAMTS2* (Table 1), and when aggregating the variants (OR = 4.06 (3.00–5.50), Table 1). In total, 59 variants were identified in these 4 genes, out of which 46 were unique, consisting of 45 missense nucleotide changes and 1 deletion of 4 nucleotides (Table S3). Twenty-three of the missense variants were predicted to be damaging or probably damaging by at least one pathogenicity algorithm (SIFT or PolyPhen2) (Table S3). The four nucleotides deletion (c.1899 + 1_1899 + 4delGTAG) was predicted to remove the complete consensus sequence of the splicing donor site in *CPAMD8* intron 15. Therefore, this allele was classified as damaging. Fifty patients (49.5%) carried at least one filtered variant in one of the four significantly enriched MMP-related genes (Table S4). Seven cases (6.9%) carried more than two variants in these genes, of which, two patients (1.9%) presented three variants (Table 2). Segregation analyses by Sanger sequencing (Figure 1 and Figure S1) revealed that these multiple variants were not associated with a typical monogenic phenotype. This was evident as biallelic variants located in the same gene were also found in one of the progenitors who did not present glaucoma (i.e., PCG103 and PCG143), showing that these mutations were carried on the same chromosome (Figure 1). While *ADAMTS2* variants appeared to segregate in an autosomal recessive compatible pattern in family PCG87, the absence of pathogenicity predicted by SIFT, PolyPhen2, and ClinVar classifications (Table S3) made it unlikely that these variants were the sole cause of the disease via a monogenic mechanism. In addition, recessive mutations of this gene are associated with Ehlers–Danlos syndrome, dermatosparaxis type (MIM # 225410). The main characteristics of this connective tissue disease, such as skin alterations, hernias, and mild to severe joint hypermobility, were not reported in the clinical records of this patient. This makes it unlikely that these mutations alone are the cause of the disease. Samples were not available for segregation analysis in one family with biallelic variants in the same gene (PCG291) and in one patient with two monoallelic variants in two genes (PCG219). Although we cannot completely dismiss the possibility that congenital glaucoma may be solely attributed to biallelic recessive *ADAMTSL4* variants, we find it improbable. This is because only one of the variants (p.Val1073Ile) is classified as damaging, probably damaging or of unknown significance, while the other (p.Gly336Glu) is predicted to be benign or likely benign (Table S3). In addition, this patient did not have clinical records of typical alterations associated with ADAMTSL4 disruption (e.g., ectopia lentis or congenital cataract). The age at diagnosis for these patients with bilateral glaucoma ranged from 5 to 144 months. Except for PCG143, all of them required surgical treatment to correct IOP (Table 2). Five patients had European ancestry, while one patient had Arab ancestry (PCG291) and another one was a sub-Saharan immigrant (PCG219).

### 2.2. Genotype–Phenotype Correlation

We also investigated a potential relationship between disease severity and the presence of rare variants in MMP-related genes. To that end, we compared two clinical parameters, namely the age at diagnosis and the average number of surgeries per eye required to control IOP, between carriers and non-carriers of filtered variants in these genes. To provide additional context, from a previous study we selected PCG patients who carried null *CYP1B1* genotypes associated with a severe phenotype characterized by early disease onset and a high number of ocular surgeries [23] (Table 3). The mean age at diagnosis did not exhibit a significant difference between carriers and non-carriers of filtered variants in MMP-related genes. However, the mean age at diagnosis for both carriers and non-carriers was higher than that of patients with null *CYP1B1* genotypes (Table 3).

Interestingly, carriers of MMP-related genes demonstrated a twofold increase in the number of glaucoma surgeries per eye compared to non-carriers (1.98 surgeries vs. 0.88 surgeries), and both groups underwent fewer surgical interventions compared to individuals with null *CYP1B1* genotypes (Table 3). There were no significant IOP differences among the three groups. Taken together, these findings suggest that patients without rare variants in MMP-related genes generally have a more favorable surgical prognosis compared to carriers of such variants, as well as those with null *CYP1B1* genotypes.

### 2.3. Expression of ADAMTSL4 in Human and Zebrafish Ocular Tissues

**Figure 2:**
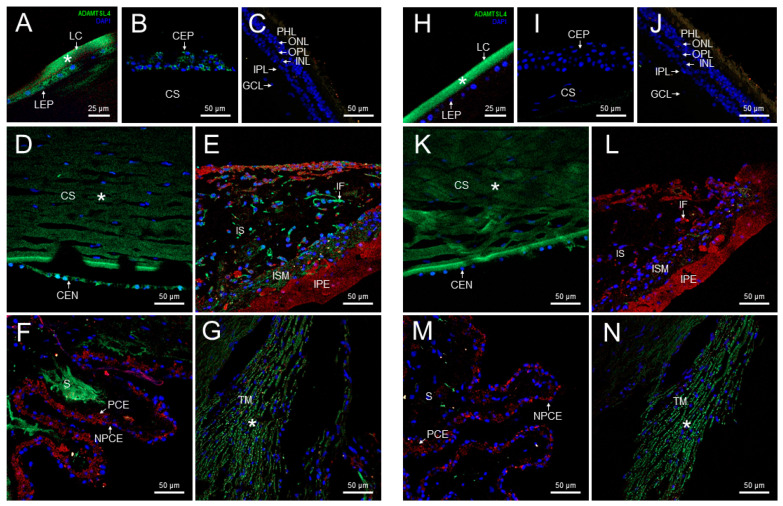
Localization of ADAMTSL4 protein in adult human ocular tissues. Fluorescent immunohistochemistry was performed on 3 μm histological sections. The sections were incubated with a primary antibody, rabbit anti-ADAMTSL4 (1:250) (MBS716409, Quimigen, Nanterre, France), followed by incubation with a green-labeled secondary antibody (Cy2-conjugate donkey anti-rabbit) (1:1000). In the resulting images, ADAMTSL4 immunoreactivity, DAPI nuclear staining and tissue autofluorescence are represented by green, blue and red signals, respectively. As a negative control, sections were incubated only with the secondary antibody (**H**–**N**). Confocal micrographs were captured to visualize the localization of ADAMTSL4 in different ocular tissues, including the lens (**A**), corneal epithelium (**B**), retina (**C**), corneal endothelium (**D**), iris (**E**), ciliary processes (**F**), and trabecular meshwork (**G**). The scale bars in panels (**B**–**G**) correspond to 50 μm, while in panel (**A**), it corresponds to 25 μm. CEN (corneal endothelium), CEP (corneal epithelium), CS (corneal stroma), GCL (ganglion cell layer), IF (iris fibroblasts), INL (inner nuclear layer), IPL (inner plexiform layer), IPE (iris pigment epithelium), IS (iris stroma), ISM (iris sphincter muscle), LC (lens capsule), LEP (lens epithelium), NPCE (non-pigmented epithelium), ONL (outer nuclear layer), OPL (outer plexiform layer), PCE (pigmented epithelium), PHL (photoreceptor layer), S (stroma), TM (trabecular meshwork). *: non-specific fluorescence.

We previously reported the expression of *CPAMD8*, one of the enriched genes, in both human and zebrafish eyes [12]. In this study, we selected another enriched gene, *ADAMTSL4*, to examine the presence of its encoded protein in glaucoma-related ocular tissues using confocal FIHC. Immunolabeling with anti-ADAMTSL4 antibodies revealed positive signals in various parts of the adult human anterior segment, including the lens epithelium and fibers (Figure 2A), corneal epithelium (Figure 2B) and endothelium (Figure 2D), as well as the stroma of the iris (Figure 2E) and ciliary processes (Figure 2F). Additionally, positive signals were observed in iris fibroblasts and iris sphincter muscle (Figure 2E). The retina was not stained with this antibody (Figure 2C). The specificity of these signals was confirmed by the absence of staining in the corresponding negative controls (Figure 2H–M). However, the negative controls revealed non-specific immunostaining in the lens capsule, corneal stroma and trabecular meshwork (Figure 2H,K,N, asterisks).

**Figure 3:**
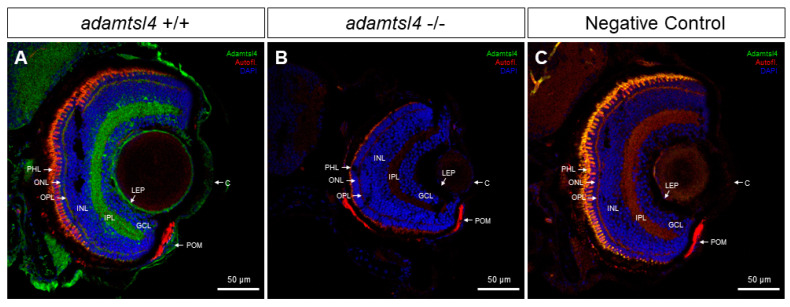
Localization of ADAMTSL4 protein in zebrafish eye larvae (6 dpf) by confocal fluorescence immunohistochemistry. Fluorescent immunohistochemistry was performed on 10 μm histological sections of zebrafish eyes from wild type (+/+) (**A**) and *adamtsl4* KO (−/−) (**B**) specimens. The sections were incubated with a rabbit anti-ADAMTSL4 primary antibody (MBS716409, Quimigen) at a dilution of 1:250, followed by a Cy2-conjugated donkey anti-rabbit secondary antibody at a dilution of 1:1000. As a negative control (**C**), a section was incubated only with the secondary antibody. In the resulting images, ADAMTSL4 immunoreactivity is represented by green signals, DAPI nuclear staining by blue signals, and tissue autofluorescence by red signals. Scale bars in the panels indicate 50 μm. The images are representative of the observed results in three zebrafish of each genotype. C: cornea. GCL: ganglion cell layer. INL: inner nuclear layer. IPL: inner plexiform layer. LEP: lens epithelium. ONL: outer nuclear layer. OPL: outer plexiform layer. PHL: photoreceptor layer. POM: periocular mesenchyme.

We also used the same anti-ADAMTSL4 antibody to investigate the ocular expression of the ortholog protein in zebrafish larvae (6 dpf) using FIHC. Consistent with the human eye, positive signals were observed in the cornea and lens epithelium of wild type zebrafish (Figure 3A). Interestingly, ADAMTSL4 immunolabeling was also detected in the periocular mesenchyme, which plays a role in the morphogenesis of iris and anterior chamber angle structures (Figure 3A). Furthermore, the antibody decorated the two retinal plexiform layers, indicating the presence of the protein in these layers. These signals were completely absent in an *adamtsl4* knockout zebrafish line and in ocular tissue sections incubated only with the secondary antibody (Figure 3B and Figure 3C, respectively), which were used as negative controls, providing robust evidence for the specificity of the signals.

### 2.4. Functional Analysis of Selected ADAMTSL4 Variants in Cells in Culture and in Zebrafish Embryos

**Figure 4:**
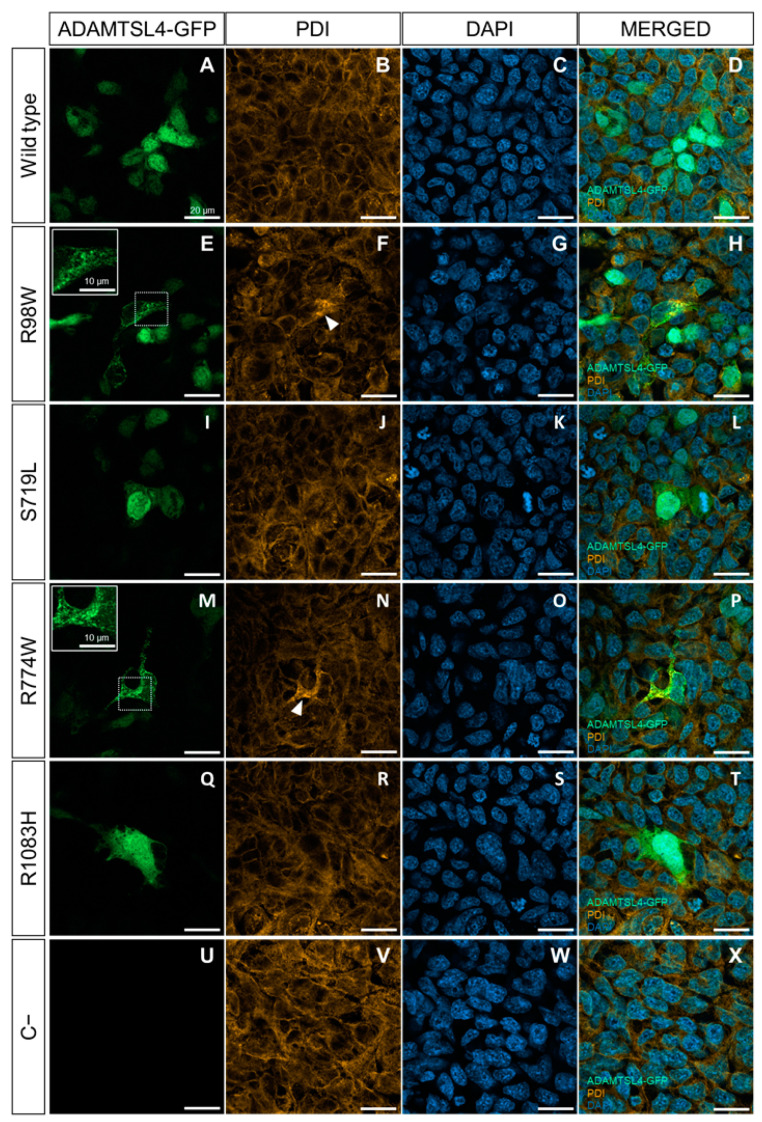
Evaluation of the functional effect of four rare *ADAMTSL4* variants identified in childhood glaucoma patients, in HEK-293T cells. Subcellular distribution of the wild type protein (**A**) and variants R98W (**E**), S719L (**I**), R774W (**M**), and R1083H (**Q**) in HEK293T Cells (green signals). The cells were transfected with cDNA constructs encoding the different variants, fused to GFP at their C-terminal as a reporter protein and analyzed 48 h after transfection. (**U**) Non-transfected cells were used as a negative control. (**B**,**F**,**J**,**N**,**R**,**V**) Immunocytochemistry was performed to detect PDI, a marker of ER stress (orange signals). (**C**,**G**,**K**,**O**,**S**,**W**) DAPI nuclear staining (blue signals). (**D**,**H**,**L**,**P**,**T**,**X**) Merged signals. The scale bars in the panels correspond to 20 μm. The white arrowheads indicate increased PDI signal colocalized with the granular expression pattern of the R98W and R774W variants. The inserts in panels (**E**,**M**) show granular deposits of recombinant protein, and dotted squares indicate the magnified areas. The images are representative of five fields per variant. Additional representative photographs are shown in Figure S3. R98W: p.Arg98Trp. S719L: p.Ser719Leu. R774W: p.Arg774Trp. R1083H: p.Arg1083His.

We selected four *ADAMTSL4* variants predicted to be pathogenic (p.Arg98Trp, p.Ser719Leu, p.Arg774Trp and p.Arg1083His) for functional evaluation using both cells in culture and zebrafish. In addition, we included the wild type sequence as a control. The variants were cloned by site-directed mutagenesis and fused to GFP at their C-terminus as a reporter molecule. We have previously used HEK-293T cells to assess the functional effects of gene variants by comparing the cellular localization of transiently overexpressed recombinant wild type and mutant proteins [37]. Upon transfection into HEK-293T cells, detection of GFP-fluorescence revealed the presence of the wild type protein in both the cytoplasm and nucleus (Figure 4A–D). In contrast, two mutant proteins (p.Arg98Trp and p.Arg774Trp) accumulated intracellularly in a granular pattern, probably within the ER, and were absent in the nucleus (Figure 4E–H and Figure 4M–P, respectively). The remaining two variants (p.Ser719Leu and p.Arg1083His) displayed expression patterns similar to those of the wild type protein (Figure 4I–L and Figure 4Q–T, respectively). The quantitative analysis showed that approximately 70–80% of cells expressing variants p.Arg98Trp and p.Arg774Trp presented intracellular accumulation of the protein (Figure S2). In parallel, immunochemical detection of PDI, a marker of ER stress [46], was performed to evaluate protein misfolding induced by these variants. Cells expressing the wild type protein showed a diffuse cytoplasmic signal, indicative of the presence of endogenous PDI in the ER (Figure 4B–D).

In contrast, cells expressing p.Arg98Trp and p.Arg774Trp showed increased PDI immunofluorescence compared to cells expressing the wild type ADAMTSL4 (Figure 4F–H and Figure 4N–P, respectively), suggesting the presence of induced ER stress. Cells expressing the other two variants (p.Ser719Leu and p.Arg1083His) showed PDI fluorescence levels similar to those of cells expressing the normal protein (Figure 4J–L and Figure 4R–T, respectively).

**Figure 5:**
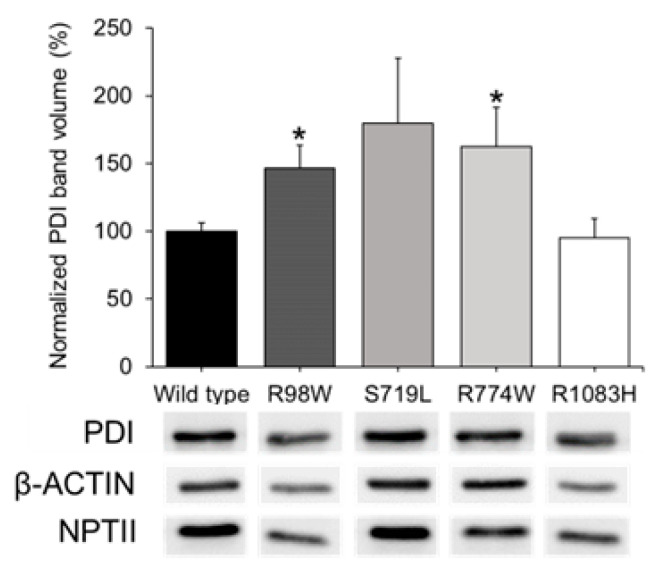
Western blot analysis of ER stress associated with the expression in HEK-293T cells of four rare ADAMTSL4 variants identified in childhood glaucoma patients. HEK-293T cells were transfected with various cDNA constructs as explained in the legend of Figure 3. Forty-eight hours after transfection, cells lysates were prepared and analyzed by Western immunoblot. PDI, beta-actin (protein loading control), and NPTII (plasmid expression control) immunosignals were quantified by densitometry. PDI levels were normalized using beta-actin and NPTII. We conducted two independent transfections per cDNA construct, each performed in triplicate. *: *p* < 0.05. R98W: p.Arg98Trp. S719L: p.Ser719Leu. R774W: p.Arg774Trp. R1083H: p.Arg1083His.

The negative control indicated the specificity of the GFP signal (Figure 4U–X). To further evaluate ER stress associated with the expression of these variants, PDI was analyzed by western blotting using cell lysates of transfected cells. Detection of beta-actin and neomycin phosphotransferase II (NPTII) were used as loading and transfection controls, respectively. PDI levels were quantified by densitometry and normalized with beta-actin and NPTII. The analysis showed significantly increased PDI levels in cells expressing p.Arg98Trp and p.Arg774Trp variants compared to cells expressing the wild type protein (Figure 5). The expressions of p.Ser719Leu and p.Arg1083His showed PDI levels that were not statistically different from those associated with the wild type sequence (Figure 5).

**Figure 6:**
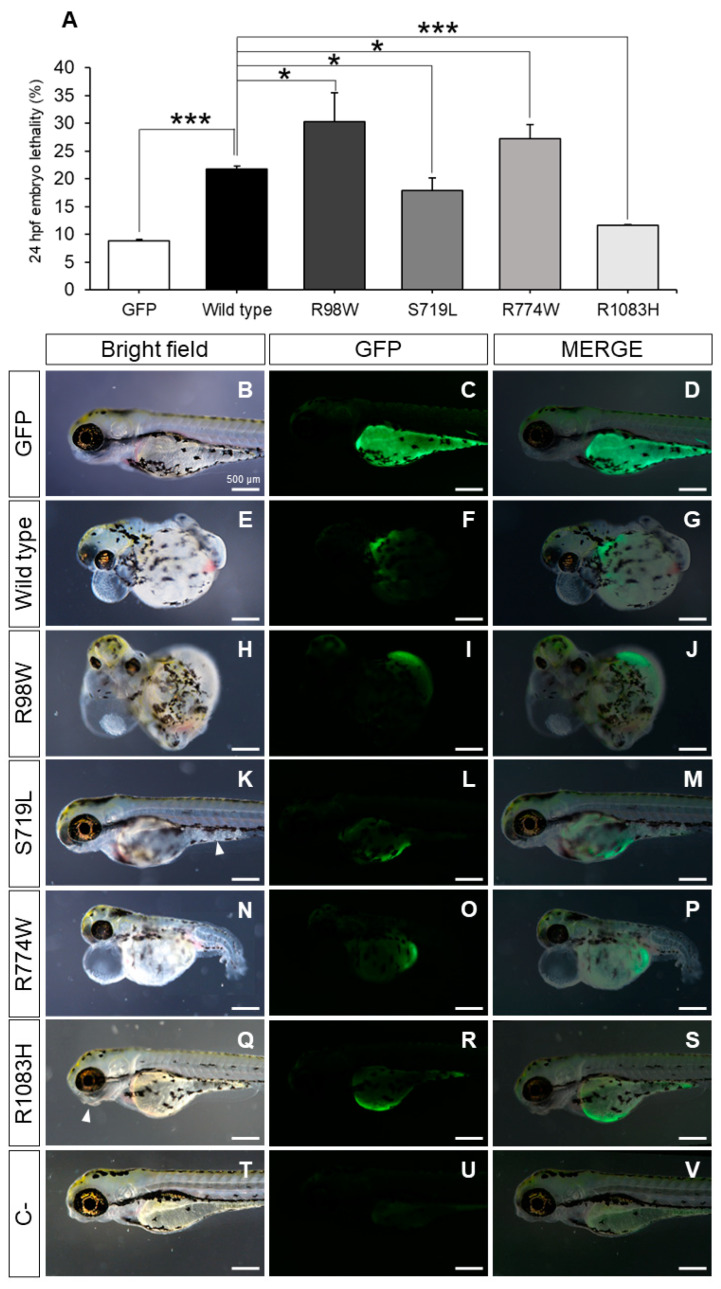
Evaluation of the functional effect of four rare ADAMTSL4 variants identified in childhood glaucoma patients, by heterologous expression in zebrafish. One-cell zebrafish embryos were microinjected with pcDNA3.1-hADAMTSL4-GFP cDNA constructs encoding the four variants (R98W, n = 374; S719L, n = 381; R774W, n = 477; and R1083H, n = 485) and the wild type protein (n = 1013). (**A**) Embryo lethality was quantified at 24 h after microinjection and (**B**–**S**) the phenotypes and recombinant protein expression were evaluated in larvae by bright field and fluorescence microscopy (3 dpf), respectively. The images are representative of the larvae expressing the recombinant proteins. Additional representative larvae are shown in Figure S4. (**T**–**V**) pcDNA3.1-GFP was microinjected as a control (n = 784). *: *p* < 0.05; ***: *p* < 0.001. R98W: p.Arg98Trp. S719L: p.Ser719Leu. R774W: p.Arg774Trp. R1083H: p.Arg1083His.

Thus, the results support the pathogenicity of variants p.Arg98Trp and p.Arg774Trp due to ADAMTSL4 misfolding, while p.Ser719Leu and p.Arg1083His do not appear to significantly disrupt the protein’s structure. As ADAMTSL proteins lack catalytic activity, an enzymatic assay to evaluate the function of this protein is not available. To overcome this limitation, we evaluated the functional impact of the mutants by comparing the effects of heterologous expression of wild type and mutant ADAMTSL4 cDNA constructs in zebrafish embryos. This experimental approach has been used to evaluate the effect of other human gene mutations [47]. Zebrafish embryos were microinjected with the different cDNA constructs. Expression of the control construct encoding wild type ADAMTSL4 resulted in early (24 hpf) lethal phenotypes in 20% of the embryos; this level of lethality was two times higher than that observed in embryos microinjected with a control cDNA construct-encoding GFP (Figure 6A). This result shows that the unregulated heterologous overexpression of wild type ADAMTSL4 is toxic to zebrafish embryos.

Zebrafish embryos expressing variants p.Arg98Trp and p.Arg774Trp exhibited a three-fold increase in lethality compared to the GFP controls and approximately one and a half times higher lethality than embryos expressing wild type ADAMTSL4 (Figure 6A). Interestingly, mutants p.Ser719Leu and p.Arg1083His led to reduced lethality compared to the wild type protein (Figure 6A), indicating that these variants induce a partial loss-of-function (hypomorphic) effect on the protein.

Control zebrafish embryos (3 dpf) microinjected with the cDNA-encoding GFP showed normal morphology and mosaic fluorescence, generally in the yolk sack (Figure 6B–D), but embryos expressing wild type ADAMTSL4 and variants p.Arg98Trp and p.Arg774Trp presented global maldevelopment and severe generalized edema, which were particularly noticeable in the pericardium and yolk sack (Figure 6E–G, Figure 6H–J and Figure 6N–P, respectively). The expression of variants p.Ser719Leu and p.Arg1083His was associated with less severe tissular alterations (Figure 6K–M and Figure 6Q–S, respectively), such as jaw maldevelopment (Figure 6Q, arrowhead, and Figure 6S) and diffuse appearance of the tissue in some areas of the body (Figure 6K, arrowhead, and Figure 6M), suggesting an alteration of the organizational pattern. These results are in accordance with a hypomorphic, or partial loss-of-function (LoF), effect associated with these variants. The morphological and tissular changes correlated with the expression of the different ADAMTSL4 proteins (Figure 6K,M). Green fluorescence was absent in non-injected zebrafish embryos (Figure 6T–V), confirming the specificity of the signals. These findings support the notion that overexpression of these proteins disrupts normal embryonic development to varying degrees, likely by altering the extracellular matrix.

### 2.5. Functional Testing in Zebrafish of the Hypothesis on the Mutation Burden of MMP-Related Genes in Glaucoma

To investigate the influence of the mutation burden detected in glaucoma patients on the disease, we evaluated the ocular phenotypes resulting from the combination of monoallelic LoF mutations in some of the enriched genes. For simplicity, we selected *adamtsl4* and *cpamd8*, which are evolutionarily conserved in zebrafish, and we took advantage of two unpublished knockout zebrafish lines that were generated in our laboratory. By crossing the *adamtsl4* and *cpamd8* knockout lines, we obtained double heterozygotes (*adamtsl4*/+ and *cpamd8*/+).

**Figure 7:**
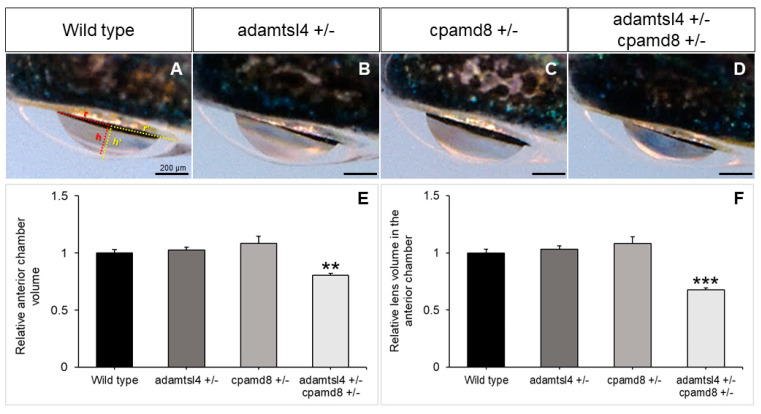
Functional interaction between zebrafish *adamtsl4* and *cpamd8* genes. (**A**–**D**) To obtain double heterozygotes (*adamtsl4*/+ and *cpamd8*/+), zebrafish progenitors with heterozygous mutations in both adamtsl4 and cpamd8 genes were crossed. The progeny were genotyped, and the ocular anterior segment was evaluated in adult specimens (2 months) using brightfield microscopy. Sibling wild type (+/+) and single heterozygous (+/−) animals were used as controls. The photographs are representative of the eyes observed. (**E**,**F**) The volumes of the anterior segment and the lens within the anterior chamber were calculated as indicated in Methods section. These calculations assumed that the anterior chamber and lens can be represented as spherical caps. In panel (**A**), the base (r) and height (h) of the spherical caps for the anterior chamber and lens are depicted in yellow and red, respectively. These values were normalized to those of wild type eyes. The following number of eyes were used: Wild type: n = 16; *adamtsl4* +/and *cpamd8* +/−: n = 16; *adamtsl4* +/− *cpamd8* +/−: n = 158. **: *p* < 0.01. ***: *p* < 0.001.

In line with the hypothesis that childhood glaucoma-causing genes play a role in ocular development, we observed a reduction in the volume of both the anterior chamber of the eye and the portion of the lens inside the anterior chamber in the double heterozygotes compared to the wild type and single heterozygous adult zebrafish (Figure 7A–D). Quantitative analysis of these volumes confirmed their similarity in wild type zebrafish and single heterozygous *adamstsl4* and *cpamd8* zebrafish. However, in the double heterozygotes, these volumes were significantly reduced by approximately four-fold (Figure 7E,F).

These findings provide strong support for the existence of a genetic interaction between these two genes and offer evidence to suggest that their combined partial functional alterations may also lead to morphological changes in the anterior chamber, potentially contributing to childhood glaucoma.

## 3. Discussion

Despite the advances in identifying genetic alterations underlying childhood glaucoma, genomic analyses assuming a monogenic inheritance of the disease have failed to identify the causative genetic alterations in many patients. This fact, along with other lines of evidence, suggests a complex mechanism of transmission that involves the participation of multiple genes. Exceptions to Mendelian expectations are also increasingly recognized in other rare diseases, likely reflecting the action of modifier genes [48,49,50]. In the present study we investigate the possible role in childhood glaucoma of rare variants in MMP-related genes. Our mutational burden analysis, applying a stringent statistical correction for multiple testing, suggests that partial functional alteration of four of these genes, combined with additional mild impairment in other genes, might contribute to the ECM anomalies present in the TM of patients affected by childhood glaucoma. Our filtering strategy selected uncommon variants potentially disrupting the function of proteins. Most of the unique variants (97.8%) were missense nucleotide changes, of which more than half were classified as damaging or probably damaging by at least one pathogenicity algorithm, providing a bioinformatic support for their functional impact. Most computational tools are developed to identify highly penetrant and clinically relevant missense variants, present in classic Mendelian diseases. However, our assumption is that the variants underlying the disease in our cohort of patients are mildly deleterious, and therefore, difficult to identify bioinformatically. Although highly time-consuming functional assays are required to evaluate the functional impact of these variants, the evidence that most rare missense alleles are deleterious in humans [51] also indicates that this group of missense variants might contribute to the genetic basis of the disease in our cohort. We hypothesize that accumulation of mild or partial functional alterations in different genes involved in ECM formation may form part of a complex pathogenic mechanism resulting in development and progression of childhood glaucoma. The identification of an increased burden of rare variants in these metalloproteinase-related genes in patients with this type of glaucoma suggests the potential significance of analyzing genetic variants within these genes. Further investigations are necessary to determine whether such analysis could be valuable in identifying individuals at a higher risk for the disease and predicting the progression of the disease. Understanding the impact of these rare variants on disease pathogenicity could guide the development of targeted therapies aimed at modulating metalloproteinase-related pathways or compensating for their dysregulation. The four enriched genes are expressed in ocular tissues [52,53,54,55] and the presence of ADAMTSL4 and CPAMD8 proteins has been demonstrated in anterior segment structures of the eye involved in glaucoma [12,55,56,57]. In addition, these genes play relevant roles in the physiology of the ocular ECM and their individual functional alterations are associated with different ocular pathologies, suggesting that the observed enrichment of rare variants in glaucoma patients may dysregulate MMP-related processes, contributing to glaucoma and other ocular pathologies. In this line, *ADAMTS2* encodes a procollagen proteinase that plays a crucial role in the cleavage of fibrillar procollagens types I-III and type V [58], involved in the recessively inherited Ehlers–Danlos syndrome type VIIC, which manifest ocular alterations, including glaucoma in a few patients [59,60]. *ADAMTS18* participates in the morphology in various tissues and organs [61] and its LoF results in abnormal ocular development [62]. *ADAMTSL4*, participates in zonular formation and maintenance [54], and is involved in ectopia lentis [63]. *CPAMD8* encodes a protein that is part of the A2M/C3 (alpha-2-macroglobulin/complement 3) protein family [64], which present a broad spectrum of endopeptidase inhibitor activity [65], and is associated with autosomal recessive anterior segment dysgenesis 8 (https://search.clinicalgenome.org/kb/genes/HGNC:23228, accessed on 27 April 2023). It has been suggested that *CPAMD8* loss-of-function may alter the regulation of extracellular proteinases involved in ECM remodeling [12].

The detection of the ADAMTSL4 protein in the ocular anterior segment of both adult human and zebrafish larvae suggests that it has evolutionary conserved and specific roles in the physiology of these ocular tissues, as well as in glaucoma. In addition, the presence of the protein in the retina of zebrafish larvae could indicate additional functions or involvement in retinal physiology beyond the anterior segment.

The intracellular accumulation, along with increased PDI expression in HEK-293T cells of two of the identified *ADAMTSL4* variants (p.Arg774Trp and p.Arg98Trp), indicated the induction of ER stress, which is associated with various pathological conditions, including glaucoma [66,67,68,69]. These results provide a potential mechanism for the role of *ADAMTSL4* variants in childhood glaucoma. The functional defect of the identified *ADAMTSL4* variants was also supported by the overexpression studies in zebrafish embryos, which revealed two different effects caused by the four variants evaluated. Variants p.Ser719Leu and p.Arg1083His were found to be less harmful than the wild type protein, suggesting a partial loss-of-function effect. On the other hand, variants p.Arg774Trp and p.Arg98Trp showed increased lethality, indicating a gain-of-function effect. We did not observe any evidence of cell death when ADAMTSL4 variants were overexpressed in HEK-293T cells. Consequently, the embryo lethality associated with overexpression of ADAMTSL4 variants is likely due to functional alterations in the extracellular matrix (ECM) that disrupt normal embryonic development, rather than a direct cytotoxic effect. Our primary interest was in identifying functional differences between the wild type and mutant forms of ADAMTSL4, rather than elucidating the mechanisms underlying these differences. To determine if the observed effects are due to alterations in cell viability, changes in the ECM or other mechanisms, further experiments are necessary.

Double heterozygous functional suppression of the zebrafish orthologs of *ADAMTSL4* and *CPAMD8*, resulted in a reduction in the volume of the anterior chamber of the adult eye, demonstrating the collaborative role of these genes in the development of anterior segment ocular structures. Moreover, this result suggests that the combined dysfunction of both genes could disrupt the normal development of the anterior segment of the eye and play a role in the pathogenesis of different ocular disorders, including glaucoma. Additional research is needed to validate these findings and to evaluate the impact on IOP resulting from the combined presence of partial functional defects in these genes.

The limitations of this investigation include the relatively small number of patients available for the association study and the assumption that additional unidentified gene variants with subtle negative impacts, which are challenging to demonstrate, are also involved in the development of glaucoma. Additionally, zebrafish may not accurately represent the nature of human ocular development and the pathogenesis of glaucoma.

## 4. Materials and Methods

### 4.1. Subjects

This study included patients enrolled from Hospital Clínico San Carlos (Madrid, Spain). A total of 101 unrelated childhood glaucoma patients (86 PCG and 15 JOAG), who did not carry pathogenic variants in *CYP1B1*, *FOXC1*, or *MYOC* genes, were selected for exome sequencing. The patients were mostly Spaniards of European ancestry (96%) and 4% were Arab and Sub-Saharan immigrants. Glaucoma specialists conducted comprehensive clinical examinations on all patients, including slit-lamp biomicroscopy, gonioscopy, biometry, intraocular pressure (IOP) measurement, and ophthalmoscopy. The childhood glaucoma diagnoses were made based on previously established criteria [23,26].

### 4.2. Human Tissue Samples

The eye of a 45-year-old Caucasian female donor, without any known ocular pathology, was obtained from the USA National Disease Research Interchange within 24 h after enucleation and processed as previously described [12]. For immunohistochemical analysis, histological microtome sections (3 µm) of the eye were used.

### 4.3. Animals

Wild type AB zebrafish (Danio rerio) were maintained under a 14 h light/10 h dark cycle at a temperature of 28 °C and fed a standard diet following established protocols [70]. Detailed experimental conditions are provided as Supplementary Methods.

### 4.4. Next-Generation Sequencing

Peripheral blood samples were collected from the subjects, and genomic DNA was extracted using the Qiagen (Germantown, MD, USA) QIAamp DNA Blood Mini Kit. Whole-exome sequencing (WES) was carried out by Macrogen using the Twist Core Exome kit (Agilent Technologies, Santa Clara, CA, USA). The libraries were pooled and sequenced on an Illumina NovaSeq6000 System (Illumina, Foster City, CA, USA) with 151 bp paired-end reads, ensuring that over 98% of the target bases had a minimum coverage of 20×. Additional experimental information is provided in Supplementary Methods.

### 4.5. Variant Prioritization

A total of 76 matrix metalloproteinase genes (Table S1) were selected for aggregate rare variant burden analysis in childhood glaucoma patients. A multistep filtering approach was implemented to identify potential disease-causing variants, as described in Supplementary Methods. The presence of variants with pathogenic genotypes (i.e., compound heterozygous or homozygous recessive variants or heterozygous dominant variants) in known glaucoma-related genes, such as *LTBP2*, *TEK*, *PITX2*, *PAX6*, *GPATCH3* and *GUCA1C*, that may explain the disease in a monogenic fashion was ruled out in all patients included in the study.

### 4.6. Fluorescence Immunohistochemistry (FIHC)

In this study, two types of tissue sections were utilized: paraffin-embedded human eye sections and zebrafish embryo cryosections. Detailed experimental conditions are provided as Supplementary Methods.

### 4.7. ADAMTSL4 Cloning and Site-Directed Mutagenesis

The human ADAMTSL4 cDNA (gene ID: 54507), cloned in the pCR4-TOPO cloning vector (Bioscience, ref: IRCBp5005E0212Q), was amplified and subcloned into an expression vector as described in Supplementary Methods. The four missense mutations (p.Arg98Trp, p.Ser719Leu, p.Arg774Trp, and p.Arg1083His) were also generated as described in Supplementary Methods, using primers shown in Table S2.

### 4.8. Expression of Recombinant Proteins in Human Cells in Culture and Analysis of Endoplasmic Reticulum Stress

Human embryonic kidney 293T cells were seeded on coverslips in 24-well plates until they reached 70–80% confluence. They were transiently transfected with the different ADAMTSL4-GFP cDNA constructs at a concentration of 1.0 μg using the Superfect Transfection Reagent (Qiagen) according to the manufacturer’s instructions. Detailed experimental conditions are provided as Supplementary Methods.

### 4.9. Evaluation of the Functional Effect of Gene Variants by Heterologous Expression in Zebrafish

Microinjection was performed on one-cell stage zebrafish embryos (50–300 embryos per experiment) using a Femtojet 5247 microinjector (Eppendorf, Hamburg, Germany) under a Nikon SMZ18 stereomicroscope. A total volume of 3 nL containing the respective pcDNA3.1-hADAMTSL4-GFP constructs (7 ng/μL) was injected. As a negative control, embryos were injected with pcDNA3.1-GFP. To ensure robustness of the results, at least three independent experiments were conducted for each cDNA construct, utilizing different zebrafish progenitors in each experiment. Lethality was quantified 24 h after microinjection and expressed as a weighted average value (%). Larval phenotypes and ADAMTSL4-GFP protein fluorescence were evaluated at 3 days post-fertilization (dpf) using a Nikon SMZ18 stereo microscope.

### 4.10. Morphological Characterization of Zebrafish Anterior Segment Phenotypes

Adult zebrafish eyes (2 months post-fertilization) were dorsally photographed using a Nikon SMZ18 stereo microscope. The volumes of the anterior chamber and the lens within the anterior chamber were measured based on the assumption that they are spherical caps as indicated in the Supplementary Methods.

## 5. Conclusions

In summary, this study offers new insights into the role of ECM genes and its functional disruption on childhood glaucoma development. Further investigations are required to elucidate the precise mechanisms through which these variants contribute to disease pathogenesis and their relevance in clinical contexts. These findings may also have wider implications for understanding the molecular mechanisms involved in other inherited and unresolved eye pathologies.

## Figures and Tables

**Table 1:** Rare variant burden analysis across four significantly enriched genes in childhood glaucoma vs. controls.

		Childhood Glaucoma Alleles (%) [n = 202]	ESP6500 * Alleles (%) [n = 8600]	gnomAD * v2.1.1 Alleles (%) [n = 104,400]	*p*-Value (Chi-Square Test) (CG vs. ESP6500)	*p*-Value (Chi-Square Test) (CG vs. gnomAD v2.1.1)	Odds Ratio (95% CI) (CG vs. ESP6500)	Odds Ratio (95% CI) (CG vs. gnomAD v2.1.1
Aggregate genes	Rare frameshift, non-sense, missense and donor/acceptor splicing sites	59 (29.20)	854 (10.03)	9489 (9.13)	<1 × 10^−15^	<1 × 10^−15^	3.49 (2.56–4.77)	4.06 (3.00–5.50)
	Rare Synonymous	17 (8.42)	773 (9.05)	10,584 (9.83)	6.39 × 10^−1^	5.77 × 10^−1^	0.86 (0.52–1.42)	0.84 (0.51–1.39)
*ADAMTS2*	Rare frameshift, non-sense, missense and donor/acceptor splicing sites	14 (6.93)	140 (1.63)	1611 (1.56)	6.52 × 10^−8^	5.00 × 10^−9^	4.49 (2.55–7.93)	4.70 (2.72–8.10)
	Rare Synonymous	2 (0.99)	184 (2.15)	2616 (2.43)	3.79 × 10^−1^	2.70 × 10^−1^	0.46 (0.11–1.85)	0.40 (0.10–1.61)
*ADAMTS18*	Rare frameshift, non-sense, missense and donor/acceptor splicing sites	14 (6.93)	178 (2.07)	2953 (2.70)	9.47 × 10^−6^	5.01 × 10^−4^	3.52 (2.01–6.18)	2.68 (1.56–4.62)
	Rare Synonymous	3 (1.49)	158 (1.84)	2110 (1.93)	9.17 × 10^−1^	8.36 × 10^−1^	0.81 (0.25–2.55)	0.76 (0.24–2.39)
*ADAMTSL4*	Rare frameshift, non-sense, missense and donor/acceptor splicing sites	16 (7.92)	176 (2.05)	2213 (2.06)	6.97 × 10^−8^	2.08 × 10^−8^	4.10 (2.41–6.99)	4.10 (2.45–6.84)
	Rare Synonymous	3 (1.49)	83 (0.97)	1545 (1.41)	7.09 × 10^−1^	8.31 × 10^−1^	1.54 (0.48–4.91)	1.05 (0.34–3.29)
*CPAMD8*	Rare frameshift, non-sense, missense and donor/acceptor splicing sites	15 (7.43)	360 (4.34)	2712 (2.84)	5.24 × 10^−2^	2.13 × 10^−4^	1.77 (1.04–3.03)	2.75 (1.62–4.65)
	Rare Synonymous	9 (4.46)	348 (4.19)	4313 (4.20)	9.92 × 10^−1^	9.94 × 10^−1^	1.07 (0.54–2.10)	1.06 (0.55–2.08)

*: individuals of European ancestry.

**Table 2:** Genetic and clinical features of patients with at least two filtered variants in significantly enriched genes.

Patient	*ADAMTS2*	*ADAMTS18*	*ADAMTSL4*	*CPAMD8*	Total Number of Variants per Patient	Age at Diagnosis (Months)	IOP at Diagnosis (mm Hg) (RE/LE)	Cup/Disc Ratio (RE/LE)	Surgical Treatment (Number of Surgeries)	Gender/Laterality
PCG67-GI	p.Glu188Gln	p.Gln146His			2	144	47/37	0.4/0.3	T (4), G/T (6), AV, G	
PCG85		p.Pro932Leu		p.Asp746Glu	2	10	NA	NA	G/G (2), T (2)	M/B
PCG87	p.Pro29Ser p.Leu23Pro				2	16	48/42	0.8/0.8	G/G	M/B
PCG103				p.Arg1862Gln p.Ala1492Pro	2	8	NA	NA	G/G	F/B
PCG143	p.Asn312Ser p.Leu23Pro			p.Arg1090Ser	3	60	NA	NA	None	F/B
PCG219			p.Pro195Leu	p.Ala1267Thr	2	5	NA	NA	T/T	F/B
PCG291			p.Val1073Ile p.Gly336Asp	p.Ile823Val	3	36	NA	NA	G/G	M/B

AV: AHMED valve. G: goniotomy. NA: not available. RE/LE: right eye/left eye. T: trabeculectomy.

**Table 3:** Clinical features of carriers of rare variants in MMP-related genes.

Variable	MMP-Related Gene Carriers (A)	MMP-Related Gene Non-Carriers (B)	PCGs with Null *CYP1B1* Genotypes (n = 37) (C)	p (A vs. B)	p (A vs. C)	p (B vs. C)
^a^ Age at diagnosis (months) (mean ± SD)	17.1 ± 37.4 (n = 50)	12.18 ± 25.2 (n = 28)	1.9 ± 5.2	ns	0.0075	0.0089
Number of surgeries per eye (mean ± SD)	1.9 ± 1.4 (n = 47)	0.8 ± 1.40 (n = 31)	3.1 ± 1.7	0.001	0.0014	0.00000019
IOP at diagnosis (mm Hg) (mean ± SD)	30.0 ± 1.4 (n = 48)	30.5 ± 9.3 (n = 30)	28.0 ± 5.5	ns	ns	ns

^a^ Only patients with a clinical diagnosis of congenital glaucoma were considered for the analysis. ns: not significant.

## Data Availability

Data are contained within the article or Supplementary Materials.

## Supplementary Materials

The following supporting information can be downloaded at: https://www.mdpi.com/article/10.3390/ijms25115757/s1.
