# Supplementary material for: The Increased Burden of Rare Variants in Four Matrix Metalloproteinase-Related Genes in Childhood Glaucoma Suggests a Complex Genetic Inheritance of the Disease
Source: Int J Mol Sci. 2024 May 25;25(11):5757. doi: 10.3390/ijms25115757 (PMC11171635; doi:10.3390/ijms25115757)
Supplement: Supplementary file 1 [file ijms-25-05757-s001.zip › ijms-2938569-supplementary.pdf]

SUPPLEMENTARY METHODS

Animals

Zebrafish embryos were raised at a temperature of 28 °C in E3 medium (pH 7.2), which consisted of 5 mM NaCl, 0.17 mM KCl, 0.33 mM $CaCl_2$, 0.33 mM $MgSO_4$, and 0.0001% methylene blue. For imaging purposes, zebrafish embryos and larvae were anesthetized with 0.02% tricaine methanesulfonate (MS 222, Sigma-Aldrich, St. Louis, MO, USA) and immobilized using a 2% methylcellulose solution. Imaging was performed using a Nikon SMZ18 microscope equipped with a Nikon DS-Ri2 camera.

Next-generation sequencing

gDNA libraries were prepared by Macrogen (Seoul, Korea) using the Twist Library Preparation EF Kit (50 ng of gDNA/sample), with full-length combinatorial dual index TruSeq-compatible Y- adapters (Illumina, Foster City, CA, USA). CD index adapters were ligated to enzymatically sheared gDNA (200 bp) and amplified by PCR. Exome capture was carried out with the Twist exome probe according to the Twist library preparation protocol (Agilent, Santa Clara, USA). Clustering and Sequencing was performed in the Novaseq6000 system (Illumina). The base calling files provided by the platform were converted into FASTQ files by the Illumina package bcl2fastq v2.20.0. Paired-end sequences were mapped to the human reference genome (hg19 from UCSC) using the mapping program BWA (v0.7.17). PCR duplicates were removed using MarkDuplicates.jar from 'Picard-tools' package. Variant genotyping was performed with Haplotype Caller of GATK (v4.0.5.1), which detects SNP and short indels candidates at nucleotide resolution. Variant Filtering (using dbSNP (138, 154) and SNPs from the 1000 genome project (Phase3) and annotation were carried out using the GATK tool (v4.0.5.1) and SnpEff (5.0e 2021-03-09), respectively. SnpEff annotated with additional databases, including ESP6500 (SI_V2), ClinVar (07/2021), dbNSFP (v4.2c) and ACMG information.

Variant Prioritization

Initially, variants present in childhood glaucoma patients that were common in the European population were filtered out based on their minor allele frequency (>1%) in the Exome Aggregation Consortium (ExAC) (https://exac.broadinstitute.org/) or gnomAD

(https://gnomad.broadinstitute.org/) databases, and variants with a genotype quality of less than 50 reads were excluded. The remaining variants included rare frameshift, nonsense, missense, and donor/acceptor splicing site variants. Rare synonymous variants were used as controls.

The initial control group was composed of variant calls from the Whole Exome Sequencing (WES) of 4,300 individuals of European ancestry, obtained from the National Heart, Lung, and Blood Institute (NHLBI) Exome Sequencing Project Exome Variant Server (Seattle, WA, USA, http://evs.gs.washington.edu/EVS/, accessed in September 2022). Additionally, we incorporated variant calls from exomes and genomes of 52,200 individuals of European ancestry from the gnomAD v2.1.1 database (https://gnomad.broadinstitute.org/, accessed in September 2022).

Chi-square tests and odds ratio analyses (with 95% confidence intervals) were conducted to identify genes exhibiting an increased burden of rare variants in patients with childhood glaucoma compared to controls. The Haldane–Anscombe correction was applied to calculate the odds ratio when one of the cells has zero value. Statistically significant values were defined as p values passing a significance level of 0.05 adjusted by Bonferroni correction to control for false discoveries in multiple testing. Sanger sequencing was conducted to confirm the presence of rare variants in the enriched genes coinciding within the same patient.

Fluorescence immunohistochemistry (FIHC)

The paraffin-embedded human eye sections (3 µm) were obtained using a Leica RM 2135 BioCut Rotary Microtome, while the zebrafish cryosections (10 µm) were obtained from 6 dpf zebrafish larvae that were fixed in 4% paraformaldehyde (PFA) in 0.1 M PBS (pH, 7.3) overnight at 4°C. The zebrafish larvae were then cryo-protected by immersion in 30% sucrose in PBS for 2 days at 4°C, embedded in 10% porcine gelatin with 15% sucrose, and stored at -80 °C. Both types of tissue sections were treated with a blocking solution composed of 10% fetal bovine serum (FBS), 1% DMSO, and 1% Triton X-100 in DPBS at room temperature for 1 h. The paraffin-embedded human eye sections and zebrafish larvae cryosections were incubated with a primary antibody, rabbit anti-

ADAMTSL4 (1:250) (MBS716409, Quimigen, Nanterre, France), overnight at 4°C. Following this, the sections were incubated with a Cy2-conjugated donkey anti-rabbit secondary antibody (1:1000) (Jackson ImmunoResearch, West Grove, PA, USA) for 1 h at room temperature. DAPI counterstaining was performed using DAPI (D8417, Sigma-Aldrich), and the samples were mounted in Fluorescence Mounting Medium (F6182, Sigma-Aldrich). A LSM710 Zeiss confocal microscope was used to visualize the samples. In human eye sections, DAPI fluorescence was recorded at 400–488 nm, Cy2-conjugated antibody fluorescence at 488–561 nm, and tissue autofluorescence at 561–700 nm. In zebrafish larvae, DAPI fluorescence was recorded at 400–472 nm, Cy2-conjugated antibody fluorescence at 490–527 nm, and tissue autofluorescence at 572–625 nm. Negative controls were created by omitting the primary antibodies. Two tissue sections per eye were used for analysis.

ADAMTSL4 cloning and site-directed mutagenesis

The human ADAMTSL4 cDNA (gene ID: 54507), cloned in the pCR4-TOPO cloning vector (Bioscience, ref: IRCBp5005E0212Q), was amplified using the specific primers incorporating NotI and EcoRV restriction cleavage sites: 5’-ATAATGCGGCCGCGTGTGTCCCCCACTG-3’ and 5’-CGGACGATATCCAGGAGGGATCCTGGGGAGACCG-3’. The resulting amplicon, containing the full-length ADAMTSL4 sequence, was inserted into the NotI-EcoRV sites of the mammalian expression vector pcDNA3.1(–) (Invitrogen, San Diego, CA, USA). This vector had previously been modified by introducing the green fluorescent protein (GFP) between the BamHI-HindIII sites. To generate missense mutations (p.Arg98Trp, p.Ser719Leu, p.Arg774Trp, and p.Arg1083His), the wild-type ADAMTSL4 DNA cloned in the pcDNA3.1 vector containing GFP was used as a template with the QuikChange site-directed mutagenesis kit (Stratagene, La Jolla, CA, USA), using specific PCR primers (Table S2). The resulting mutated cDNAs were subcloned into the NotI-EcoRV sites of the modified pcDNA3.1(–) mammalian expression vector as described above. All DNA constructs obtained were sequenced to confirm accurate insertion and the absence of unintended mutations.

Expression of recombinant proteins in human cells in culture and analysis of endoplasmic reticulum stress

After 48 hours of transfection with the different ADAMTSL4-GFP cDNA constructs, cells were fixed with 4% paraformaldehyde for 10 min and incubated with a blocking solution containing 10% FBS, 1% DMSO, and 1% Triton X-100 in DPBS at room temperature for 1h. Subsequently, for endoplasmic reticulum (ER) stress analysis the cells were incubated overnight at 4°C with a rabbit anti-protein disulfide isomerase (PDI) primary antibody (1:200) (P7496, Sigma-Aldrich), followed by incubation with a Cy3-conjugated donkey anti- rabbit secondary antibody (1:1000) (Jackson ImmunoResearch, West Grove, USA) at room temperature for 1 h. After staining with DAPI (D8417, Sigma-Aldrich), the cells were mounted on coverslips using Fluorescence Mounting Medium (F6182, Sigma-Aldrich) and subsequently examined under an LSM710 Zeiss confocal microscope. The fluorescence emitted by DAPI, ADAMTSL4-GFP, and the Cy3-conjugated antibody was registered at wavelengths of 400–495 nm, 495–540 nm, and 540–700 nm, respectively. Negative controls were prepared by omitting the primary antibodies.

For ER stress quantification, cells were treated with 100 µl of lysis buffer containing proteinase inhibitors. The collected cells were vortexed at maximum speed for 30 s, incubated on ice for 30 min, and sonicated for 10 s with a cycle of 0.5 s. SDS-PAGE, using a 10% polyacrylamide gel, was conducted utilizing the Mini-PROTEAN III gel electrophoresis system (Bio-Rad, Hercules, CA, USA). For western blot analysis, samples were normalized for protein content using the BCA Protein Assay Kit (Pierce, Rockford, IL, USA), and cell lysates containing 80 µg of total protein were treated with loading buffer containing β- mercaptoethanol, followed by 5 min boiling and subjected to SDS-PAGE. After protein separation, Hybond ECL nitrocellulose membranes (Amersham, Uppsala, Sweden) were used to transfer the samples for subsequent immunodetection. PDI was detected using a rabbit anti-PDI primary antibody (1:500) (P7496, Sigma) overnight at 4°C, followed by incubation with a goat anti-rabbit secondary antibody (1:1000) (1858415, Pierce) for 1 h at room temperature. Beta-actin was used as a loading control and detected by incubating with a primary mouse anti-beta-actin antibody (1:1000) (GT5512, GeneTex, San Antonio, CA, USA) overnight at 4ºC, followed by incubation with a goat anti-mouse secondary antibody (1:1000) (32430, Invitrogen) for

1 h at room temperature. Neomycin phosphotransferase II (NPTII) was employed as a transfection control and was detected using a mouse anti-NPTII primary antibody (1:250) (4B4D1, Novus, Littleton, CO, USA) overnight at 4 ºC, followed by incubation with a goat anti-mouse secondary antibody (1:1000) (32430, Invitrogen) for 1 h at room temperature. Chemiluminescence detection was performed using SuperSignal West Dura reagents (ThermoScientific/Pierce, Rockford, IL, USA). Densitometry analysis was performed to quantify protein bands. Scanned films were analyzed using Quantity One 4.6.6. analysis software (Bio-Rad) in at least three independent experiments conducted in triplicate.

Morphological characterization of zebrafish anterior segment phenotypes

The formula used for calculation of volumes of the anterior chamber and the lens within the anterior chamber was $V=(1/3)\pi h^2(3R-h)$, where h represents the height of the cap and R denotes the radius of the sphere. The radius of the sphere was calculated using the formula $R=(r^2+h^2)/(2h)$, with r being the radius of the base.

Statistics

Statistical comparisons between experimental groups were performed using either the *t*-test or the one-way ANOVA using the SigmaStat 2.0 software (SPSS Science Inc.).

SUPPLEMENTARY FIGURES

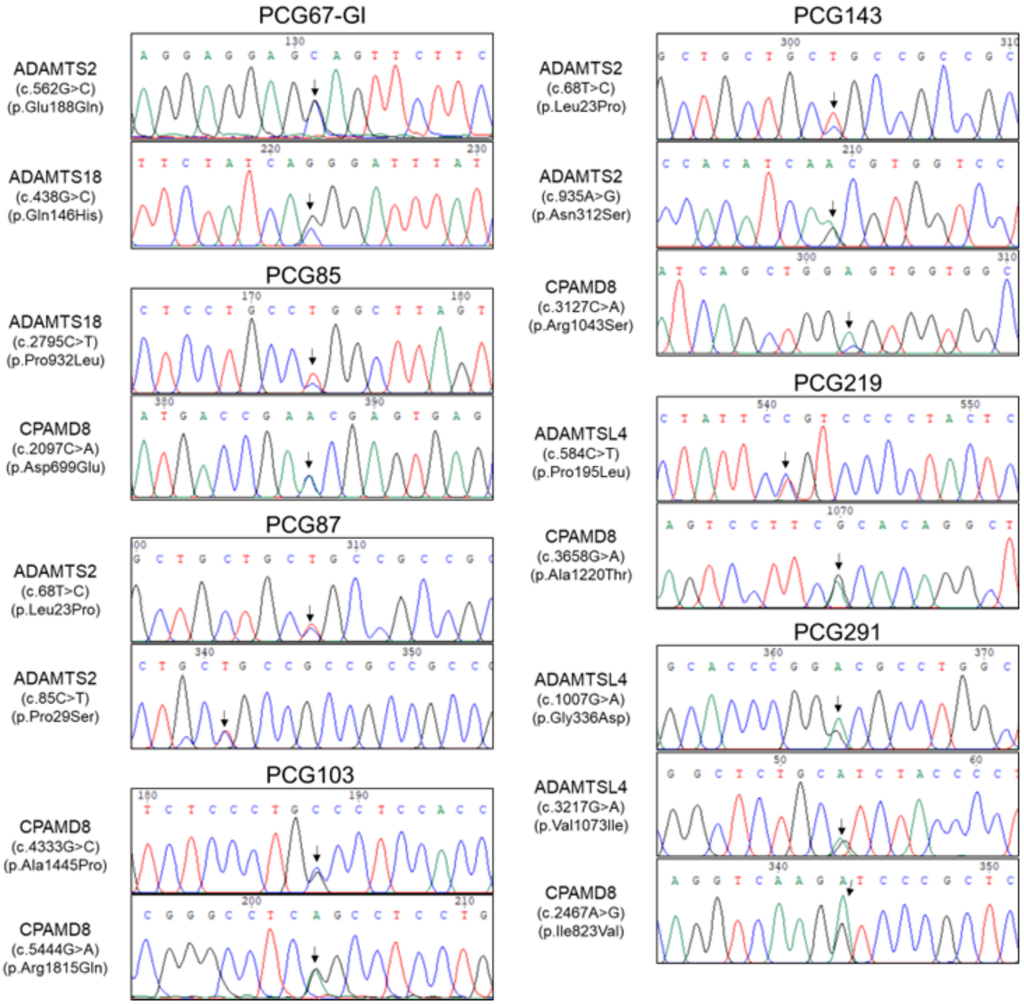

Figure S1. Electropherograms of variants identified in childhood glaucoma patients who carry more than one variant in MMP-related genes (Figure 1). Arrows in the electropherograms indicate the location of variants.

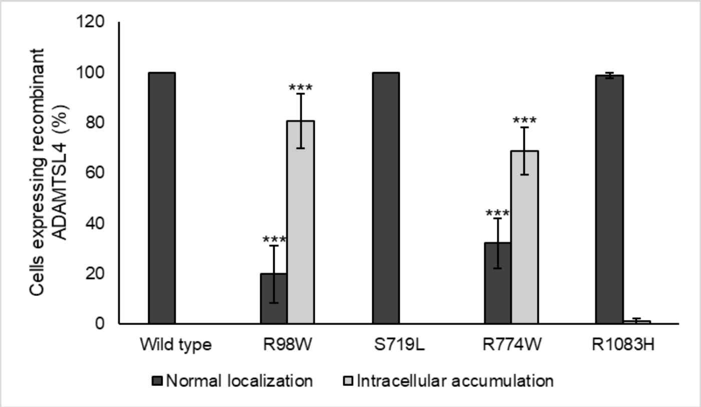

Figure S2. Quantification of the altered subcellular localization of different recombinant ADAMTSL4 variants expressed in HEK-293T cells. The cells were transfected with cDNA constructs encoding the different variants, as described in Figure 4. The percentage of cells with normal or altered subcellular localization was calculated from images similar to those shown in Figure 4. Seven fields per variant (350-400 total cells/variant) were used to calculate the percentages. The wild type protein was localized in the cytoplasm and nucleus (normal localization), while the altered subcellular distribution was characterized by intracellular accumulation in a granular pattern and absence nuclear signal (Figure 4). The values for each variant were compared with those of the wild type protein. ***: $p<0.001$.

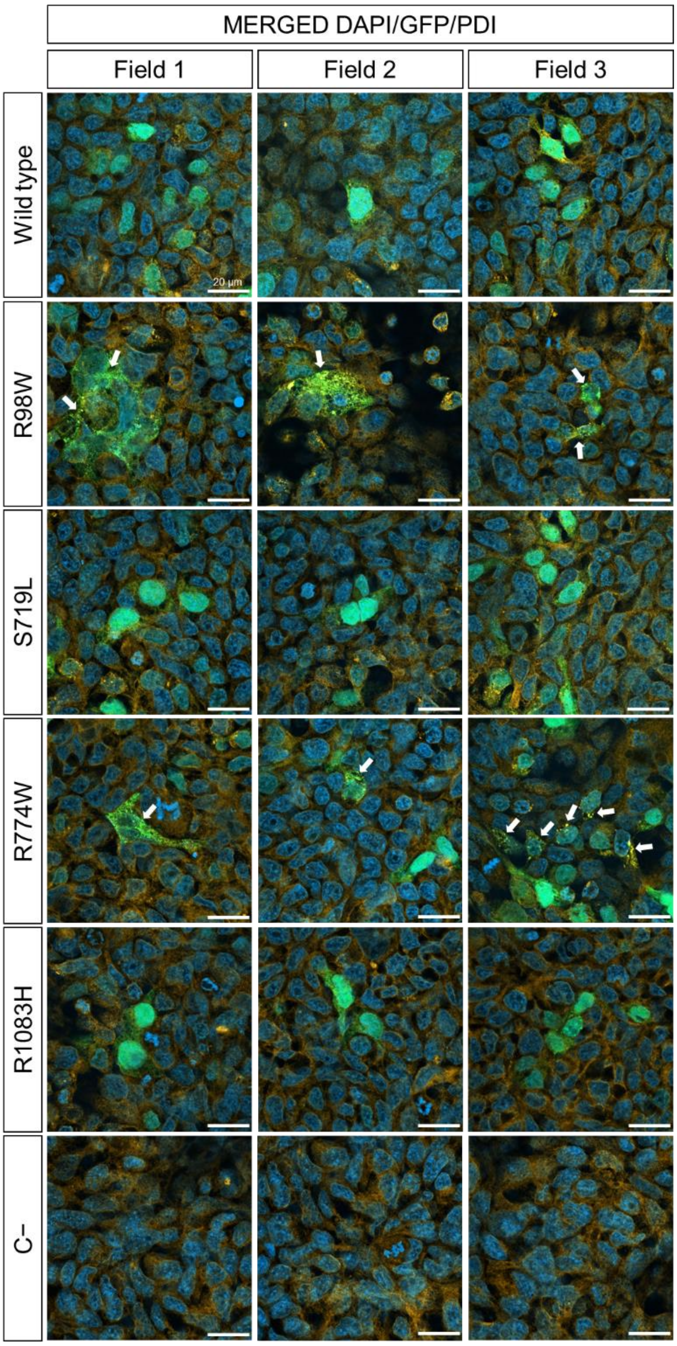

Figure S3. Evaluation of the functional effect of four rare *ADAMTSL4* variants identified in childhood glaucoma patients, in HEK-293T cells. Representative photographs, additional to those shown in Figure 4, are provided. The cells were transfected with cDNA constructs encoding the different variants, fused to GFP at their C-terminal as a reporter protein and analyzed 48 h after transfection. The white arrowheads indicate increased PDI signal colocalized with the granular expression pattern. The scale bars in the panels correspond to 40 µm. There are three different fields per variant. R98W: p.Arg98Trp. S719L: p.Ser719Leu. R774W: p.Arg774Trp. R1083H: p.Arg1083His.

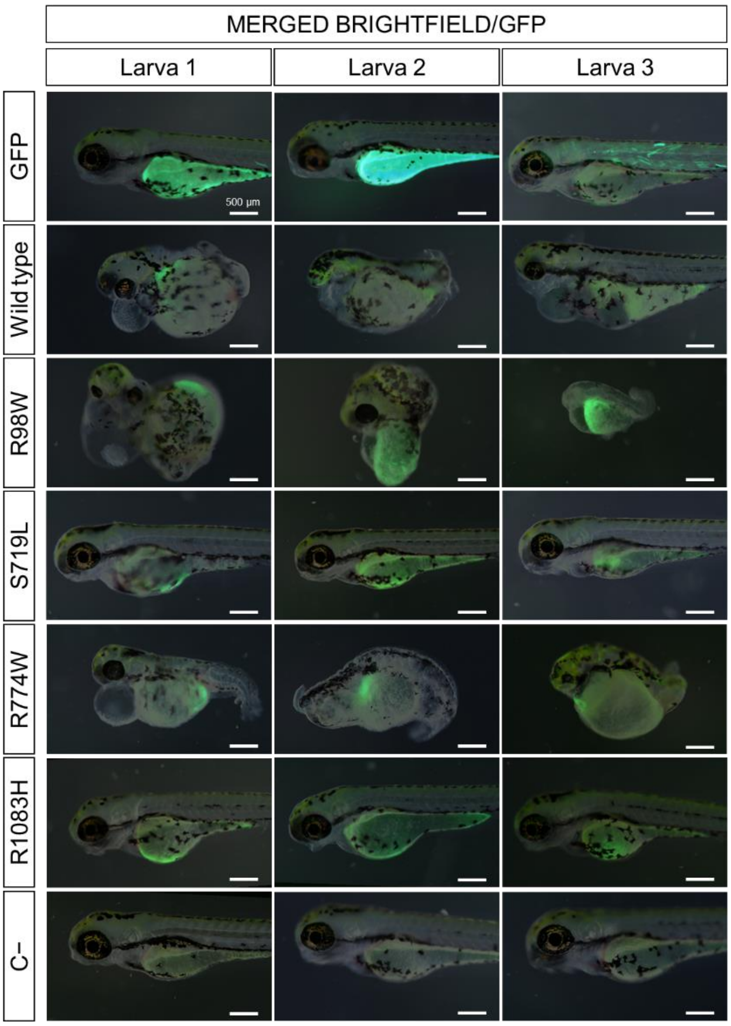

Figure S4. Evaluation of the functional effect of four rare ADAMTSL4 variants identified in childhood glaucoma patients, by heterologous expression in zebrafish. Representative photographs, in addition to those presented in Figure 6, are included. Please note, that the images in the first column are identical to those in Figure 6. R98W: p.Arg98Trp. S719L: p.Ser719Leu. R774W: p.Arg774Trp. R1083H: p.Arg1083His. The expression of variants p.Ser719Leu and p.Arg1083His was associated with less severe tissular alterations, suggesting a partial loss-of-function effect.

Table S1. Frequency of rare variants in 76 matrix metalloproteinase-related genes in childhood glaucoma patients and controls. *significant p-values after conservative Bonferroni correction for multiple testing ($p<6.57x10^{-4}$) are shown in bold. The Haldane–Anscombe correction was applied to calculate the odds ratio when one of the cells has zero value.

Genes	Childhood glaucoma Alleles [n=202] [n (%)]	ESP6500 glaucoma Alleles [n=8600] [n (%)]	gnomAD v2.1.1 Alleles (%) [104400]	p-Value (Chi-square test) (CG vs. ESP6500)	p-Value (Chi-square test) (CG vs. gnomAD v2.1.1)	Odds Ratio (95% CI) (CG vs. ESP6500)	Odds Ratio (95% CI) (CG vs. gnomAD v2.1.1)
ADAM2	3 (1.49)	248 (2.89)	3676 (3.45)	3.32E-01	1.82E-01	0.51 (0.16-1.60)	0.42 (0.13-1.32)
ADAM7	5 (2.48)	111 (1.29)	1286 (1.18)	2.53E-01	1.68E-01	1.94 (0.78-4.80)	2.13 (0.87-5.18)
ADAM8	7 (3.47)	132 (1.54)	2166 (2.34)	6.04E-02	4.10E-01	2.29 (1.06-4.97)	1.50 (0.70-3.19)
ADAM9	4 (1.98)	114 (1.33)	1688 (1.57)	6.25E-01	8.55E-01	1.50 (0.55-4.11)	1.26 (0.47-3.41)
ADAM10	2 (0.99)	21 (0.25)	451 (0.44)	1.77E-01	5.20E-01	4.07 (0.95-17.47)	2.26 (0.56-9.11)
ADAM11	3 (1.49)	62 (0.72)	1008 (1.00)	4.02E-01	7.30E-01	2.08 (0.65-6.67)	1.50 (0.48-4.69)
ADAM12	3 (1.49)	91 (1.06)	1831 (1.69)	8.13E-01	9.58E-01	1.41 (0.44-4.49)	0.88 (0.28-2.75)
ADAM15	0 (0.00)	129 (1.50)	1829 (1.73)	1.45E-01	1.07E-01	0.16 (0.01-2.60)	0.14 (0.01-2.25)
ADAM17	5 (2.48)	147 (1.71)	2480 (2.27)	5.82E-01	9.66E-01	1.46 (0.59-3.59)	1.09 (0.45-2.65)
ADAM18	8 (3.96)	218 (2.54)	3104 (2.93)	3.00E-01	5.08E-01	1.58 (0.77-3.25)	1.37 (0.67-2.78)
ADAM19	2 (0.99)	307 (3.57)	3656 (3.61)	7.57E-02	7.11E-02	0.27 (0.07-1.09)	0.27 (0.07-1.08)
ADAM20	2 (0.99)	122 (1.42)	1822 (1.65)	8.31E-01	6.48E-01	0.69 (0.17-2.82)	0.60 (0.15-2.41)
ADAM21	4 (1.98)	80 (0.93)	1218 (1.11)	2.51E-01	3.98E-01	2.15 (0.78-5.92)	1.80 (0.67-4.86)
ADAM22	4 (1.98)	53 (0.64)	803 (0.76)	6.27E-02	1.12E-01	3.13 (1.12-8.72)	2.64 (0.98-7.13)
ADAM23	2 (0.99)	194 (2.26)	2614 (2.51)	3.35E-01	2.47E-01	0.43 (0.11-1.76)	0.39 (0.10-1.56)
ADAM28	6 (2.97)	153 (1.78)	2290 (2.13)	3.25E-01	5.57E-01	1.69 (0.74-3.86)	1.41 (0.62-3.18)
ADAM29	5 (2.48)	194 (2.26)	2449 (2.27)	9.71E-01	9.64E-01	1.10 (0.45-2.70)	1.09 (0.45-2.65)
ADAM30	1 (0.50)	123 (1.43)	1680 (1.55)	4.15E-01	3.52E-01	0.34 (0.05-2.46)	0.32 (0.04-2.25)
ADAM32	3 (1.49)	253 (3.10)	1713 (1.83)	2.66E-01	9.17E-01	0.47 (0.15-1.48)	0.81 (0.26-2.53)
ADAM33	6 (2.97)	50 (0.58)	1402 (1.51)	**2.00E-04***	1.60E-01	5.22 (2.21-12.32)	1.99 (0.88-4.50)
ADAMDEC1	3 (1.49)	93 (1.08)	1289 (1.18)	8.40E-01	9.38E-01	1.38 (0.43-4.39)	1.26 (0.40-3.96)
ADAMTS1	8 (3.96)	170 (1.98)	2844 (2.71)	8.42E-02	3.82E-01	2.04 (0.99-4.21)	1.48 (0.73-3.00)
ADAMTS2	14 (6.93)	140 (1.63)	1611 (1.56)	**6.52E-08***	**5.00E-09***	4.49 (2.55-7.93)	4.70 (2.72-8.10)
ADAMTS3	4 (1.98)	173 (2.01)	2149 (1.96)	8.25E-01	8.12E-01	0.98 (0.36-2.68)	1.01 (0.37-2.72)

Genes	Childhood glaucoma Alleles [n=202] [n (%)]	ESP6500 glaucoma Alleles [n=8600] [n (%)]	gnomAD v2.1.1 Alleles (%) [104400]	p-Value (Chi-square test) (CG vs. ESP6500)	p-Value (Chi-square test) (CG vs. gnomAD v2.1.1)	Odds Ratio (95% CI) (CG vs. ESP6500)	Odds Ratio (95% CI) (CG vs. gnomAD v2.1.1)
ADAMTS4	5 (2.48)	193 (2.24)	3357 (3.14)	9.83E-01	7.37E-01	1.11 (0.45-2.72)	0.78 (0.32-1.91)
ADAMTS5	5 (2.48)	54 (0.63)	1244 (1.19)	6.20E-03	1.74E-01	4.01 (1.59-10.12)	2.11 (0.87-5.14)
ADAMTS6	2 (0.99)	86 (1.00)	1603 (1.46)	7.31E-01	7.94E-01	0.99 (0.24-4.05)	0.68 (0.17-2.72)
ADAMTS8	11 (5.45)	240 (2.87)	4973 (4.94)	5.32E-02	8.64E-01	1.95 (1.05-3.63)	1.11 (0.60-2.04)
ADAMTS9	7 (3.47)	266 (3.10)	2289 (2.10)	5.96E-02	2.71E-03	2.31 (1.06-5.02)	3.43 (1.59-7.39)
ADAMTS10	6 (2.97)	164 (1.91)	3889 (3.73)	4.10E-01	7.02E-01	1.57 (0.69-3.60)	0.79 (0.35-1.78)
ADAMTS12	7 (3.47)	215 (2.50)	1169 (1.06)	5.26E-01	2.80E-03	1.40 (0.65-3.01)	3.36 (1.58-7.15)
ADAMTS13	6 (2.97)	154 (1.79)	1457 (1.50)	3.32E-01	1.53E-01	1.68 (0.73-3.84)	2.01 (0.89-4.54)
ADAMTS14	7 (3.47)	293 (3.41)	3566 (3.30)	8.79E-01	9.45E-01	1.02 (0.47-2.18)	1.05 (0.49-2.23)
ADAMTS15	4 (1.98)	320 (3.73)	3286 (3.11)	2.64E-01	4.70E-01	0.52 (0.19-1.41)	0.63 (0.23-1.69)
ADAMTS16	5 (2.48)	126 (1.51)	1723 (1.62)	4.17E-01	4.92E-01	1.65 (0.67-4.09)	1.54 (0.64-3.76)
ADAMTS17	6 (2.97)	70 (0.82)	1397 (1.31)	4.00E-03	7.89E-02	3.71 (1.59-8.65)	2.30 (1.02-5.20)
ADAMTS18	14 (6.93)	178 (2.07)	2953 (2.70)	**9.47E-06***	**5.01E-04***	3.52 (2.01-6.18)	2.68 (1.56-4.62)
ADAMTS19	3 (1.49)	49 (0.57)	1157 (1.16)	2.26E-01	9.20E-01	2.63 (0.81-8.50)	1.28 (0.41-4.02)
ADAMTS20	4 (1.98)	69 (0.80)	1024 (1.00)	1.53E-01	3.00E-01	2.49 (0.90-6.90)	1.99 (0.74-5.37)
ADAMTSL1	5 (2.48)	213 (2.51)	1776 (1.62)	8.46E-01	4.97E-01	0.99 (0.40-2.42)	1.54 (0.63-3.74)
ADAMTSL2	3 (1.49)	35 (0.42)	798 (1.02)	9.04E-02	7.62E-01	3.54 (1.08-11.59)	1.46 (0.47-4.58)
ADAMTSL3	4 (1.98)	56 (0.65)	1042 (0.95)	6.63E-02	2.52E-01	3.08 (1.11-8.58)	2.11 (0.78-5.68)
ADAMTSL4	16 (7.92)	176 (2.05)	2213 (2.06)	**6.97E-08***	**2.08E-08***	4.10 (2.41-6.99)	4.10 (2.45-6.84)
ADAMTSL5	8 (3.96)	102 (1.19)	2567 (2.94)	1.50E-03	5.15E-01	3.42 (1.64-7.12)	1.36 (0.67-2.77)
BMP1	5 (2.48)	139 (1.62)	1723 (1.58)	5.03E-01	4.62E-01	1.54 (0.63-3.81)	1.58 (0.65-3.84)
MMP1	3 (1.49)	113 (1.31)	1886 (1.69)	9.19E-01	9.65E-01	1.13 (0.36-3.59)	0.88 (0.28-2.74)
MMP2	3 (1.49)	48 (0.56)	923 (0.83)	2.12E-01	5.27E-01	2.69 (0.83-8.70)	1.80 (0.57-5.63)
MMP3	1 (0.50)	159 (1.85)	1068 (0.98)	2.47E-01	7.32E-01	0.26 (0.04-1.89)	0.50 (0.07-3.59)
MMP7	0 (0.00)	21 (0.24)	303 (0.28)	9.80E-01	9.36E-01	0.98 (0.06-16.30)	0.89 (0.06-14.23)
MMP8	6 (2.97)	114 (1.33)	1393 (1.31)	9.34E-02	7.91E-02	2.27 (0.99-5.23)	2.30 (1.02-5.19)
MMP9	2 (0.99)	145 (1.70)	2284 (2.30)	6.20E-01	3.14E-01	0.58 (0.14-2.35)	0.42 (0.11-1.71)
MMP10	3 (1.49)	138 (1.61)	2057 (1.92)	8.86E-01	8.50E-01	0.92 (0.29-2.92)	0.77 (0.25-2.42)
MMP11	5 (2.48)	98 (1.14)	1852 (1.74)	1.58E-01	5.97E-01	2.20 (0.89-5.46)	1.43 (0.59-3.48)

Genes	Childhood glaucoma Alleles [n=202] [n (%)]	ESP6500 glaucoma Alleles [n=8600] [n (%)]	gnomAD v2.1.1 Alleles (%) [104400]	p-Value (Chi-square test) (CG vs. ESP6500)	p-Value (Chi-square test) (CG vs. gnomAD v2.1.1)	Odds Ratio (95% CI) (CG vs. ESP6500)	Odds Ratio (95% CI) (CG vs. gnomAD v2.1.1)
MMP13	1 (0.50)	64 (0.75)	1065 (0.98)	9.96E-01	7.34E-01	0.66 (0.09-4.80)	0.50 (0.07-3.59)
MMP14	0 (0.00)	44 (0.51)	1081 (1.05)	6.07E-01	2.64E-01	0.47 (0.03-7.74)	0.23 (0.01-3.74)
MMP15	2 (0.99)	217 (2.53)	2030 (1.98)	2.47E-01	4.48E-01	0.39 (0.10-1.56)	0.49 (0.12-1.99)
MMP16	0 (0.00)	35 (0.41)	423 (0.42)	7.32E-01	7.05E-01	0.60 (0.04-9.75)	0.58 (0.04-9.39)
MMP17	5 (2.48)	210 (2.44)	3053 (3.07)	8.40E-01	7.73E-01	1.01 (0.41-2.49)	0.80 (0.33-1.95)
MMP19	3 (1.49)	116 (1.35)	1904 (1.73)	8.85E-01	1.00E+00	1.10 (0.35-3.49)	0.85 (0.27-2.67)
MMP20	8 (3.96)	297 (3.46)	3678 (3.30)	8.49E-01	7.43E-01	1.15 (0.56-2.36)	1.21 (0.60-2.45)
MMP21	3 (1.49)	104 (1.22)	1537 (1.62)	9.85E-01	8.98E-01	1.22 (0.39-3.89)	0.92 (0.29-2.87)
MMP24	5 (2.48)	80 (0.95)	1068 (1.05)	7.12E-02	1.01E-01	2.64 (1.06-6.59)	2.39 (0.98-5.83)
MMP25	7 (3.47)	135 (1.57)	1879 (1.91)	6.81E-02	1.75E-01	2.24 (1.04-4.86)	1.85 (0.87-3.93)
MMP26	1 (0.50)	114 (1.33)	1707 (1.53)	4.75E-01	3.63E-01	0.37 (0.05-2.66)	0.32 (0.04-2.29)
MMP27	2 (0.99)	139 (1.62)	1758 (1.58)	6.75E-01	7.00E-01	0.61 (0.15-2.47)	0.62 (0.16-2.52)
MMP28	1 (0.50)	108 (1.29)	1131 (1.19)	4.98E-01	5.58E-01	0.38 (0.05-2.74)	0.41 (0.06-2.95)
PAPLN	9 (4.46)	150 (1.75)	3548 (3.37)	9.80E-03	5.13E-01	2.62 (1.32-5.21)	1.34 (0.68-2.61)
TIMP1	2 (0.99)	38 (0.56)	1058 (1.54)	7.53E-01	7.31E-01	1.76 (0.42-7.35)	0.64 (0.16-2.58)
TIMP2	0 (0.00)	10 (0.12)	128 (0.12)	5.68E-01	5.91E-01	2.02 (0.12-34.59)	2.08 (0.13-33.53)
TIMP3	0 (0.00)	6 (0.07)	116 (0.11)	3.23E-01	5.64E-01	3.26 (0.18-58.15)	2.19 (0.14-35.33)
TIMP4	1 (0.50)	7 (0.08)	319 (0.29)	4.57E-01	9.00E-01	6.08 (0.75-49.69)	1.68 (0.24-12.04)
TLL1	1 (0.50)	82 (0.95)	1017 (0.94)	7.66E-01	7.67E-01	0.52 (0.07-3.73)	0.52 (0.07-3.73)
TLL2	8 (3.96)	138 (1.61)	1661 (1.51)	2.11E-02	1.03E-02	2.52 (1.22-5.22)	2.69 (1.33-5.47)
CPAMD8	15 (7.43)	360 (4.34)	2712 (2.84)	5.24E-02	**2.14E-04***	1.77 (1.04-3.03)	2.75 (1.62-4.65)
A2M	4 (1.98)	71 (0.85)	1012 (0.96)	1.83E-01	2.58E-02	2.37 (0.86-6.55)	2.09 (0.78-5.64)
SERPINA1	0 (0.00)	135 (1.57)	2036 (1.91)	1.32E-01	8.48E-02	0.15 (0.01-2.49)	0.13 (0.01-2.04)

Table S2. Primers used to generate four *ADAMTSL4* variants by site-directed mutagenesis. Fw: forward. Rv: reverse. The dark background indicates the mutant nucleotide.

Variant	Primer	Sequence
**p.Arg98Trp**	Fw	5'-CCTCCTCCCCTGGGGCCAGGGT-3'
	Rv	5'-ACCCTGGCCCCAGGGGAGGAGG-3'
**p.Ser719Leu**	Fw	5'-CTCTGCATCTCCCGTGAGTTGGGAGAGGAA-3'
	Rv	5'-TTCCTCTCCCAACTCACGGGAGATGCAGAG-3'
**p.Arg774Trp**	Fw	5'-CGCCAGCTGCAGTGCTGGCAGGAATTTGGG-3'
	Rv	5'-CCCAAATTCCTGCCAGCACTGCAGCTGGCG-3'
**p.Arg1083His**	Fw	5'-AGCCACCTGTTGCCACTCTTGCGCACATG-3'
	Rv	5'-CATGTGCGCAAGAGTGGCAACAGGTGGCT-3'

Table S3. Rare Variants in 4 enriched matrix metalloproteinase-related genes in childhood glaucoma patients. (T: Tolerated; B: benign; D: deleterious; PD: possibly/probably damaging). ND: not described. NA: not available. US: uncertain significance. LB: likely benign. *: individuals of European ancestry.

	Base	Amino Acid	Alleles [n=202] [n (%)]	ESP6500* Alleles [n=8600] [n (%)]	gnomAD* Alleles [n=104400] [n (%)]	dbSNP ID	SIFT	Poly Phen2	ClinVar
ADAMTS2	c.68T>C	L23P	5 (2.48)	ND	135 (0.94)	rs565885690	T (0.11)	B (0)	US/B/LB
	c.85C>T	P29S	1 (0.50)	ND	ND	NA	B (0.29)	B (0)	NA
	c.110C>T	A37V	1 (0.50)	ND	5 (0.0075)	rs1057524401	T (0.68)	B (0)	US
	c.562G>C	E188Q	1 (0.50)	2 (0.023)	26 (0.020)	rs146064587	D (0)	PD (0.999)	US
	c.721C>T	R241C	1 (0.50)	2 (0.023)	4 (0.0031)	rs140045997	D (0.04)	B (0.183)	US
	c.748G>A	A250T	1 (0.50)	6 (0.070)	124 (0.096)	rs143764421	B (0.18)	B (0.003)	US/LB
	c.935A>G	N312S	1 (0.50)	ND	9 (0.0070)	rs528367185	D (0.01)	PD (0.82)	US
	c.1074A>T	E358D	1 (0.50)	ND	ND	NA	NA	NA	NA
	c.2719G>A	A907T	1 (0.50)	0 (0.00)	26 (0.023)	rs199617528	B (0.62)	B (0.007)	US
	c.3551C>T	P1184L	1 (0.50)	1 (0.011)	8 (0.0062)	rs150989902	T (0.27)	B (0.041)	US/LB
ADAMST18	c.438G>C	Q146H	2 (0.99)	6 (0.070)	213 (0.16)	rs151326659	B (0.12)	B (0.302)	LB
	c.637C>T	R213W	3 (1.49)	82 (0.95)	1273 (0.99)	rs35701343	D (0.04)	B (0.009)	B
	c.767G>A	R256Q	1 (0.50)	ND	0 (0.00)	rs775057284	D (0.01)	B (0.17)	US
	c.794C>T	P265L	1 (0.50)	0 (0.00)	0 (0.00)	rs147049816	B (0.09)	B (0.049)	US
	c.2480G>T	R827L	1 (0.50)	7 (0.081)	114 (0.088)	rs149031657	B (0.19)	B (0.006)	US
	c.2541G>T	M847I	1 (0.50)	3 (0.035)	31 (0.024)	rs114018664	D (0.03)	B (0.003)	B
	c.2599A>C	T867P	2 (0.99)	ND	4 (0.0037)	rs774518027	B (0.15)	PD (0.559)	NA
	c.2795C>T	P932L	1 (0.50)	ND	2 (0.0018)	rs765223011	D (0.05)	PD (0.714)	US
	c.3157C>T	R1053W	1 (0.50)	16 (0.19)	279 (0.22)	rs148703569	D (0)	PD (0.776)	US/B
	c.3565G>A	V1189I	1 (0.50)	57 (0.66)	881 (0.69)	rs61749042	B (0.24)	B (0.009)	B
ADAMTSL4	c.292C>T	R98W	1 (0.50)	25 (0.29)	251 (0.20)	rs148393631	D (0.01)	PD (0.653)	US/B/LB
	c.584C>T	P195L	2 (0.99)	0 (0.00)	7 (0.0054)	rs139990606	D (0.01)	B (0.174)	US/B/LB
	c.622G>A	G208S	1 (0.50)	0 (0.00)	5 (0.0039)	rs115104834	B (0.65)	B (0.007)	B
	c.1007G>A	G336D	1 (0.50)	15 (0.18)	105 (0.086)	rs142354320	B (1)	B (0)	B/LB
	c.2156C>T	S719L	1 (0.50)	2 (0.023)	99 (0.079)	rs115937511	D (0)	PD (0.856)	NA
	c.2320C>T	R774W	1 (0.50)	0 (0.00)	8 (0.0064)	rs371399521	D (0)	PD (0.997)	US
	c.2321G>A	R774Q	1 (0.50)	ND	4 (0.0032)	rs753302240	D (0)	PD (0.974)	US

	Base	Amino Acid	Alleles [n=202] [n (%)]	ESP6500* Alleles [n=8600] [n (%)]	gnomAD* Alleles [n=104400] [n (%)]	dbSNP ID	SIFT	Poly Phen2	ClinVar
	c.2357C>G	P786R	1 (0.50)	0 (0.00)	607 (0.48)	rs56055939	D (0.01)	PD (0.914)	NA
	c.2549C>T	P850L	1 (0.50)	1 (0.012)	13 (0.010)	rs146452550	B (0.14)	B (0.037)	US
	c.2807G>A	R936H	1 (0.50)	1 (0.012)	36 (0.029)	rs144358124	B (0.37)	B (0.005)	US
	c.2907C>G	N969K	1 (0.50)	0 (0.00)	3 (0.0023)	rs370834300	D (0.01)	B (0.405)	US
	c.3217G>A	V1073I	1 (0.50)	ND	14 (0.011)	rs201941243	D (0)	PD (0.83)	US
	c.3248G>A	R1083H	3 (1.49)	6 (0.070)	102 (0.080)	rs147697821	D (0.02)	PD (0.949)	LB
CPAMD8	c.182C>T	T61M	3 (1.49)	21 (0.25)	330 (0.27)	rs186779378	B (0.28)	B (0)	NA
	c.808G>A	V270I	1 (0.50)	4 (0.049)	102 (0.079)	rs201964890	B (0.15)	B (0.025)	LB
	c.1740G>C	E580D	1 (0.50)	3 (0.036)	89 (0.069)	rs138079023	D (0.03)	B (0.339)	NA
	c.1758+1_ 1758+4del	c.1758+1_ 1758+4del GTAG	1 (0.50)	ND	19 (0.015)	rs577740063	NA	NA	NA
	c.2097C>A	D699E	1 (0.50)	3 (0.036)	26 (0.020)	rs61746640	D (0)	B (0.283)	B
	c.2467A>G	I823V	1 (0.50)	5 (0.060)	28 (0.027)	rs61741636	B (0.88)	B (0.007)	NA
	c.3127C>A	R1043S	1 (0.50)	13 (0.16)	137 (0.11)	rs143655652	B (0.65)	B (0.109)	US
	c.3658G>A	A1220T	1 (0.50)	4 (0.048)	47 (0.037)	rs57249432	B (0.06)	PD (0.996)	B
	c.3997C>T	R1333C	1 (0.50)	ND	1 (0.0010)	rs757640318	D (0)	PD (0.945)	NA
	c.4333G>C	A1445P	1 (0.50)	ND	1 (0.00088)	rs1482611581	D (0.01)	PD (0.891)	NA
	c.4562C>T	P1521L	1 (0.50)	0 (0.00)	4 (0.0033)	rs200142695	B (0.38)	B (0)	US
	c.4960G>C	E1654Q	1 (0.50)	20 (0.24)	295 (0.29)	rs144048982	B (0.05)	B (0.11)	LB
	c.5444G>A	R1815Q	1 (0.50)	ND	0 (0.00)	rs893071602	T (0.23)	B (0)	NA

Table S4. Filtered variants in significantly enriched genes per patient.

Patient	*ADAMTS2*	*ADAMTS18*	*ADAMTSL4*	*CPAMD8*	Total number of Variants per patient
**PCG17**		p.Arg1053Trp			1
**PCG19**			p.Arg1083His		1
**PCG22**			p.Arg1083His		1
**PCG37**		p.Pro265Leu			1
**PCG39**				p.Thr108Met	1
**PCG42**	p.Leu23Pro				1
**PCG47**			p.Arg774Trp		1
**PCG60**	p.Glu358Asp				1
**PCG65**	p.Ala37Val				1
**PCG66**	p.Arg241Cys				1
**PCG67**	p.Glu188Gln	p.Gln146His			2
**PCG74**		p.Thr867Pro			1
**PCG78**				p.Glu1701Gln	1
**PCG85**		p.Pro932Leu		p.Asp746Glu	2
**PCG87**	p.Pro29Ser<br>p.Leu23Pro				2
**PCG88**				p.Thr108Met	1
**PCG93**				p.Pro1568Leu	1
**PCG96**			p.Arg98Trp		1
**PCG101**			p.Ser719Leu		1
**PCG103**				p.Arg1862Gln<br>p.Ala1492Pro	2
**PCG119**		p.Met847Ile			1
**PCG128**			p.Pro195Leu		1
**PCG129**		p.Thr867Pro			1
**PCG130**				c.1899+1_1899+4delGTAG	1
**PCG132**		p.Arg827Leu			1
**PCG143**	p.Asn312Ser<br>p.Leu23Pro			p.Arg1090Ser	3
**PCG174**		p.Arg213Trp			1
**PCG180**		p.Arg213Trp			1
**PCG181**				p.Val317Ile	1
**PCG193**		p.Val1189Ile			1
**PCG196**	p.Leu23Pro				1
**PCG209**				p.Glu627Asp	1
**PCG216**		p.Arg256Gln			1
**PCG219**			p.Pro195Leu	p.Ala1267Thr	2
**PCG228**			p.Pro850Leu		1
**PCG240**	p.Ala250Thr				1
**PCG252**			p.Arg936His		1

Patient	*ADAMTS2*	*ADAMTS18*	*ADAMTSL4*	*CPAMD8*	Total number of Variants per patient
**PCG262**			p.Arg1083His		1
**PCG267**	p.Ala907Thr				1
**PCG270-1**		p.Arg213Trp			1
**PCG284-1**				p.Arg1333Cys	1
**PCG290-1**			p.Asn969Lys		1
**PCG291-1**			p.Val1073Ile<br>p.Gly336Asp	p.Ile823Val	3
**PCG293-1**	p.Leu23Pro				1
**PCG294-1**	p.Pro1184Leu				1
**PCG301**			p.Gly208Ser		1
**JOAG64**			p.Arg774Gln		1
**JOAG65**		p.Gln146His			1
**JOAG67**			p.Pro786Arg		1
**MOC4**				p.Thr61Met	1
**Total number of Variants per gene**	14	14	16	15	59
